# *Drosophila* Learn Opposing Components of a Compound Food Stimulus

**DOI:** 10.1016/j.cub.2014.05.078

**Published:** 2014-08-04

**Authors:** Gaurav Das, Martín Klappenbach, Eleftheria Vrontou, Emmanuel Perisse, Christopher M. Clark, Christopher J. Burke, Scott Waddell

**Affiliations:** 1 Centre for Neural Circuits and Behaviour, University of Oxford, Tinsley Building, Mansfield Road, Oxford OX1 3SR, UK; 2 Laboratorio de Neurobiología de la Memoria, Facultad de Ciencias Exactas y Naturales, IFIBYNE-CONICET, Universidad de Buenos Aires, Buenos Aires C1428EGA, Argentina; 3 Department of Neurobiology, University of Massachusetts Medical School, 364 Plantation Street, Worcester, MA 01605, USA

## Abstract

Dopaminergic neurons provide value signals in mammals and insects [1–3]. During *Drosophila* olfactory learning, distinct subsets of dopaminergic neurons appear to assign either positive or negative value to odor representations in mushroom body neurons [4–9]. However, it is not known how flies evaluate substances that have mixed valence. Here we show that flies form short-lived aversive olfactory memories when trained with odors and sugars that are contaminated with the common insect repellent DEET. This DEET-aversive learning required the MB-MP1 dopaminergic neurons that are also required for shock learning [7]. Moreover, differential conditioning with DEET versus shock suggests that formation of these distinct aversive olfactory memories relies on a common negatively reinforcing dopaminergic mechanism. Surprisingly, as time passed after training, the behavior of DEET-sugar-trained flies reversed from conditioned odor avoidance into odor approach. In addition, flies that were compromised for reward learning exhibited a more robust and longer-lived aversive-DEET memory. These data demonstrate that flies independently process the DEET and sugar components to form parallel aversive and appetitive olfactory memories, with distinct kinetics, that compete to guide learned behavior.

## Highlights

• Flies trained with unpalatable sugar learn the sweet-, nutrient, and bad-taste qualities
• Distinct dopamine neurons reinforce the positive and negative memories in parallel
• Early conditioned aversion switches to longer-lasting nutrient-memory-guided attraction
• Flies remember individual qualities of a complex food source

## Teaser

Das et al. show that flies trained with unpalatable sugar learn both the nutritional and bad-taste qualities. Opposing memories are reinforced in parallel by distinct dopaminergic neurons. Conditioned behavior is initially aversive but soon switches to approach led by the longer-lasting nutrient-reinforced appetitive memory.

## Results and Discussion

DEET has been reported to drive aversive behavior in flies through olfactory [10–12] and gustatory [13] pathways. We therefore used a low concentration presented in solid medium (1% agar) to decrease the effects of volatile DEET and increase the chance that flies would taste and perhaps ingest it. To further encourage flies to sample DEET, we increased its palatability by adding it to a mixture of sweet sugars—3 M xylose and 100 mM sucrose (from here on referred to as “carrier”). Xylose is detected by sweet-sensitive gustatory neurons and is palatable to flies, but it contributes no measurable nutrient value [14]. The low concentration of sweet and nutritious sucrose was added to further increase palatability [14]. We first determined the optimum DEET concentration by adding increasing amounts to sugar carrier and conditioning hungry flies by pairing the exposure of the second of two odors with DEET presentation.

**Figure 1:**
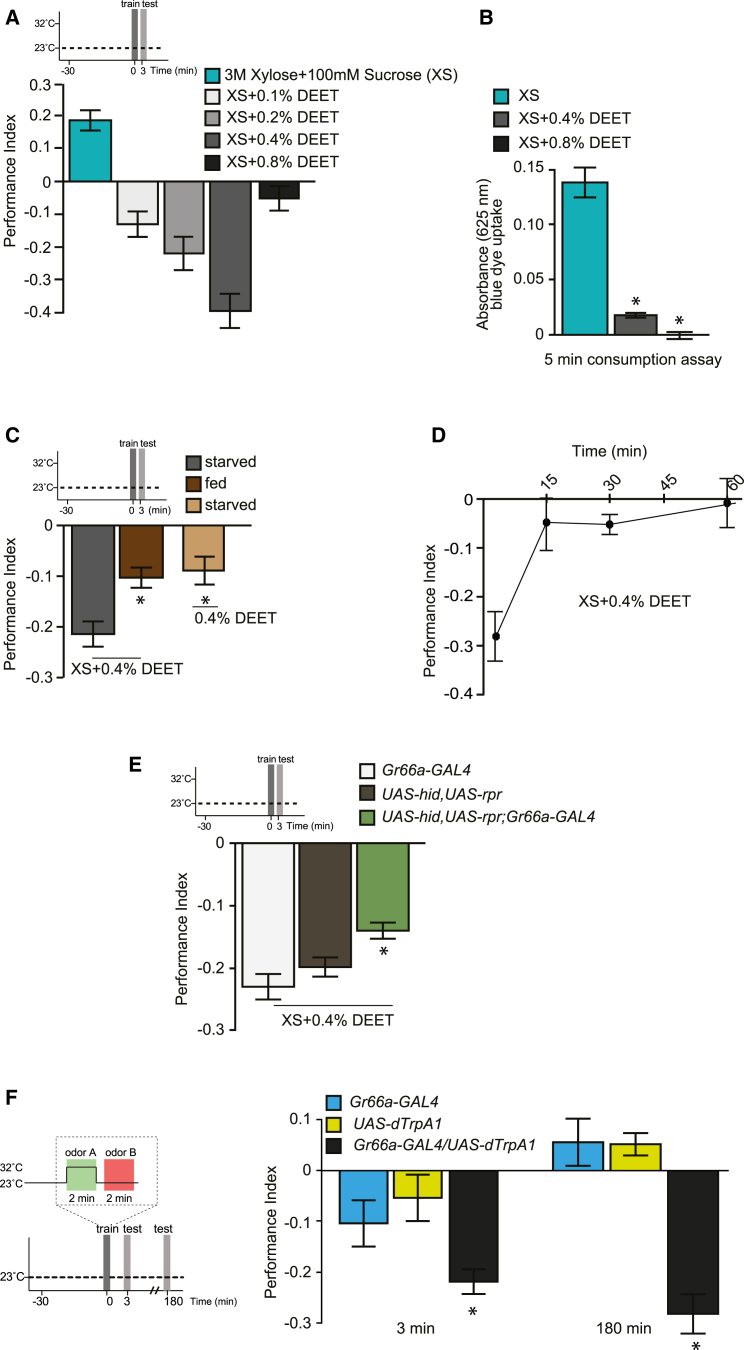
Aversive Olfactory Learning with DEET Reinforcement (A) Learning with DEET depends on concentration. Wild-type flies starved overnight were trained with 0%–0.8% DEET with a sugar carrier (3 M xylose plus 100 mM sucrose). Aversive memory performance increased with DEET concentrations up to 0.4% but was negligible with 0.8% DEET. Learning with 0.4% DEET is significantly different from that with both 0.1% and 0.8% DEET (both p < 0.001). All other group wise comparisons are not significant (all p > 0.05). ANOVA followed by Tukey’s multiple comparison test was performed. p values are multiplicity adjusted (ANOVA). n ≥ 5. (B) DEET inhibits ingestion. The amount of sugar carrier plus DEET (0.4% or 0.8%) ingested in 5 min was quantified using FD&C Blue No. 1 dye supplementation. Flies consumed sugar carrier alone, but inclusion of 0.4% or 0.8% DEET significantly reduced consumption. However, flies consumed statistically significant amounts of dye presented with 0.4% DEET, but not with 0.8% DEET (p = 0.002 and p = 0.8427, respectively, from zero; one-sample t test, n ≥ 5). (C) DEET learning is most robust when flies ingest. Hungry flies display robust immediate aversive memory with 0.4% DEET presented with sugar carrier. However, both satiated flies and those trained with 0.4% DEET without sugar carrier exhibited significantly less aversive memory performance (both p < 0.05, ANOVA, n ≥ 10). (D) DEET memory is labile. DEET reinforced memory decayed rapidly and was not significant 15 min after training (p < 0.05 versus 3 min performance and p > 0.9 versus 30 and 60 min, ANOVA, n ≥ 6). (E) Ablation of bitter-taste neurons impairs DEET learning. Flies expressing UAS-*hid* and UAS-*rpr* in *Gr66a*-GAL4 cells were trained with 0.4% DEET in sugar carrier (3 M xylose and 100 mM sucrose) and were immediately tested for memory performance. The performance of these flies was statistically different than that of the control groups (p < 0.05, ANOVA, n ≥ 23). (F) Aversive memory can be implanted with bitter-taste neuron activation contingent with odor presentation. Flies were trained by pairing dTrpA1-mediated activation of *Gr66a*-GAL4 bitter gustatory neurons with odor as shown in the schematic. Significant aversive memory was formed in *Gr66a*-GAL4/UAS-*dTrpA1* flies that persisted for at least 3 hr (at both time points p < 0.005, ANOVA, n ≥ 8). Data are shown as mean ± SEM. Asterisks denote significant difference between marked group and the relevant controls. See also Figure S1.

Flies trained with only the sugar carrier showed a significant appetitive memory (Figure 1A). In contrast, those trained with increasing amounts of DEET formed aversive memory, with the score rising in line with the increase in DEET concentration, up to 0.4%. Surprisingly, flies trained with 0.8% DEET did not exhibit significantly negative aversive memory scores, suggesting a change in the flies’ perception of DEET at this concentration. We therefore tested the effect of 0.4% and 0.8% DEET on fly feeding by measuring ingestion marked with blue food dye (Figure 1B). Whereas flies ate significant amounts of food containing sugar carrier, both 0.4% and 0.8% DEET strongly suppressed feeding behavior. However, whereas flies ate a measurable amount of dye with 0.4% DEET, ingestion was abolished with 0.8% DEET. These data suggest that the failure to train flies with 0.8% DEET reflects an inhibition of sampling by the proboscis and perhaps ingestion of DEET and sugar. To further test a requirement for feeding in learning, we attempted to train flies that were not hungry or with 0.4% DEET without sugar carrier (Figure 1C). Both of these conditions significantly impaired aversive learning when compared to hungry flies trained with 0.4% DEET in sugar carrier. We also observed a similar concentration-dependent aversive memory formation when flies were trained with bitter-tasting quinine that was mixed with sugar carrier (Figure S1A available online). Furthermore, flies that were defective in the IR40a olfactory route of DEET detection displayed normal DEET learning (Figure S1B). We therefore conclude that robust learning with 0.4% DEET-laced sugar requires the flies to attempt to eat DEET and that low DEET concentrations convert the conditioned approach that is formed when flies are trained with the sugar carrier into a conditioned aversion.

We next measured the persistence of DEET memory by conditioning flies and testing their odor preference at extended times after training (Figure 1D). Whereas aversive memory performance was robust immediately after training, no statistically significant performance was evident 15 min later. Aversive memory formed with 0.4% DEET is therefore surprisingly labile. DEET and quinine can be sensed by bitter-taste neurons [13, 15, 16], and ablation of bitter-sensing neurons with *Gr66a*-GAL4-directed expression of cell-death genes [17] partially impaired DEET, but not sugar, learning (Figures 1E and S1D). We therefore tested whether flies could be aversively conditioned by pairing odor presentation with artificial bitter-taste neuron [16, 18] activation, achieved by expression of UAS-*dTrpA1* (Figure 1F). The dTrpA1 gene encodes a transient receptor potential (TRP) channel that conducts Ca^2+^ and depolarizes neurons when flies are exposed to temperature >25°C [19]. *Gr66a*-GAL4, UAS-*dTrpA1*, and *Gr66a*-GAL4; UAS-*dTrpA1* flies were conditioned by presentation of the first odor with activating 32°C and were immediately tested for memory. *Gr66a*-GAL4; UAS-*dTrpA1* flies exhibited aversive memory that was statistically different from that of all other groups (Figure 1F). However, unlike flies conditioned with DEET (Figure 1D), significant memory remained 3 hr after training (Figure 1F). The differing persistence could result from artificial stimulation of bitter neurons being stronger than DEET activation, in addition to lacking plausible competition from a copresented sugar stimulus.

**Figure 2:**
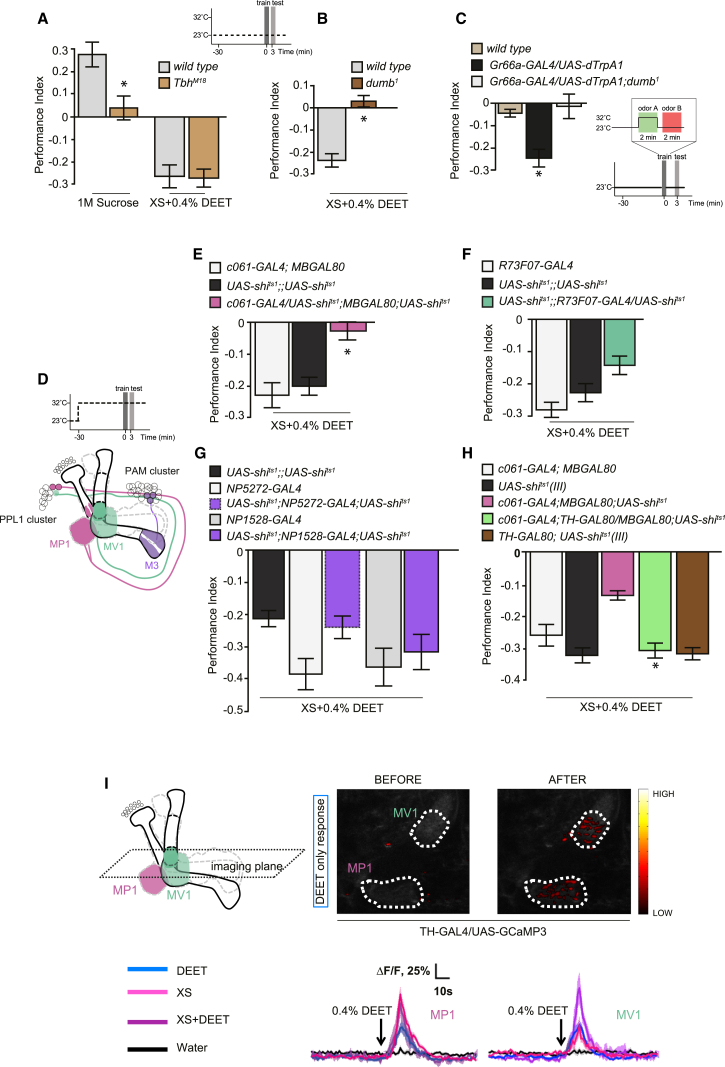
DEET Reinforcement Involves Specific Dopaminergic Neurons (A) DEET learning does not require octopamine. Appetitive memory formation with 1 M sucrose was significantly impaired in *Tbh*^*M18*^ flies (p < 0.05, t test, n ≥ 4), whereas aversive memory with DEET was statistically indistinguishable from that of control flies (p > 0.5, t test, n ≥ 6). (B) DEET memory formation was significantly impaired in *dumb*^1^ mutant flies (p < 0.001, t test, n ≥ 10). (C) Aversive memory formation with bitter-taste neuron activation is impaired in *dumb*^1^ mutant flies. Performance of *Gr66a*-GAL4/UAS-*dTrpA1* flies was statistically different from that of *Gr66a*-GAL4/UAS-*dTrpA1*; *dumb*^1^ and wild-type flies (both p < 0.005, ANOVA, n ≥ 11). (D) Schematic of the training paradigm for testing the role of the specific MP1, MV1, and M3 dopaminergic neurons in DEET learning. The innervation zone of each type of dopaminergic neuron on the ipsilateral mushroom body lobe is illustrated. MP1 and MV1 neuron cell bodies reside in the PPL1 cluster, whereas M3 is in the PAM cluster. (E) Blockade of the MP1 neurons with c061; MBGAL80; UAS-*shi*^ts1^ significantly impaired DEET learning (p < 0.001, ANOVA, n ≥ 12). (F) Blockade of the MV1 neurons with R73F07; UAS-*shi*^ts1^ did not significantly impair DEET learning (p < 0.05 versus R73F07, but p > 0.05 versus UAS-*shi*^ts1^, ANOVA, n ≥ 20). (G) Blockade of the M3 neurons with NP5272; UAS-*shi*^ts1^ or NP1528; UAS-*shi*^ts1^ did not significantly impair DEET learning (p > 0.05, ANOVA, n ≥ 7). (H) Removal of *shi*^ts1^ transgene expression from dopaminergic neurons in c061-GAL4; MBGAL80; UAS-*shi*^ts1^ (III) flies, by inclusion of *TH*-GAL80, significant restores DEET learning (p < 0.001 versus c061-GAL4; MBGAL80; UAS-*shi*^ts1^ flies). Further, performance of c061-GAL4; *TH*-GAL80/MB-GAL80; UAS-*shi*^ts1^ flies was indistinguishable from that of other control groups (all p > 0.5, ANOVA, n ≥ 8). (I) Feeding of 0.4% DEET in water evokes an increase in intracellular Ca^2+^ in MP1 and MV1 neurons, measured using UAS-*GCaMP3* expression driven by *TH*-GAL4. A time course of DEET-evoked GCaMP3 responses (ΔF/F) in MP1 and MV1 processes in the mushroom body lobes, measured at the indicated regions of interest (dashed outlines), is shown. Averaged traces are shown as solid lines, and shaded areas represent the SEM. The arrow indicates the onset of DEET presentation. Inset panels show pseudocolored activity maps of neural responses before and after DEET presentation, overlaid on grayscale images of baseline fluorescence. Additionally, DEET in carrier (3 M xylose and 100 mM sucrose) and carrier alone evoke a significant increase in intracellular Ca^2+^ in MP1 and MV1 neurons. Water presentation produced a negligible response. Average traces (from seven to 17 repetitions in three flies per condition) are shown for all groups, except 0.4% DEET by itself. Data are shown as mean ± SEM. See also Figures S2 and S3.

Octopamine is required to convey the reinforcing effects of sweet taste [9]. We therefore tested DEET learning in *Tbh*^*M18*^ mutant flies that cannot synthesize octopamine [20] (Figure 2A). Whereas appetitive conditioning with 1 M sucrose was significantly impaired in *Tbh*^*M18*^ flies, aversive learning with 0.4% DEET was indistinguishable from that of wild-type flies. Therefore, octopamine is not required for DEET learning.

Electric-shock-reinforced aversive memory formation also requires specific dopaminergic neurons and the DopR1 dopamine receptor [5–7, 21]. We therefore first determined whether DEET learning required the DopR1 receptor (Figure 2B). Mutant *dumb*^1^ flies that are defective for the DopR1 dopamine receptor did not display aversive learning with DEET. Similarly, aversive learning with artificial activation of bitter-taste neurons was abolished in *dumb*^1^ flies (Figure 2C).

The MB-MP1, MB-MV1, and MB-M3 classes of dopamine neuron have been previously implicated in shock learning [6, 7] (Figure 2D). To test whether either of these neurons were required for DEET learning, we expressed the dominant temperature-sensitive UAS-*shibire*^ts1^ transgene [22] in MP1, MV1, and M3 neurons using the c061; MBGAL80, R73F07, and NP5272 and NP1528 GAL4 drivers [7, 23, 24], respectively. The *shi*^ts1^ transgene permitted blockade of the respective neurons by performing DEET conditioning experiments at the restrictive temperature of 31°C. This analysis revealed significantly impaired DEET learning performance when MP1 neurons were blocked (Figure 2E) but nonsignificant effects when either MV1 (Figure 2F) or M3 (Figure 2G) neurons were compromised. Blockade of MP1 neurons, however, did not significantly affect DEET avoidance in naive flies (Figure S2). To further support a role for the dopaminergic MP1 neurons in c061; MBGAL80, we removed them from the expression pattern by including a *TH*-GAL80 transgene [25]. When the remaining cells were blocked during conditioning, flies exhibited levels of DEET learning that were indistinguishable from those of wild-type flies (Figure 2H). We therefore conclude that MP1 neurons are critical for DEET learning, whereas MV1 and M3 neurons contribute a lesser role. We note that prior work implicated the MV1 and M3 neurons in the formation of more persistent forms of shock-reinforced aversive memory [6, 7].

We next used live imaging to determine whether DEET ingestion activated the MP1 dopamine neurons. We expressed UAS-*GCaMP3* [26] in dopaminergic neurons with *TH*-GAL4 [27] and imaged DEET-evoked changes in fluorescence in the dopaminergic neuron processes on the mushroom body (Figure 2I). These analyses revealed strong activation of the MP1 innervated heel and MV1 innervated junction regions of the mushroom body while presenting flies with both 0.4% DEET in sugar carrier, sugar carrier alone, and DEET alone. In comparison, water presentation did not activate the MP1 and MV1 neurons. Therefore, functional imaging does not reveal obvious valence specificity of MP1 and MV1 signals, being activated by both sugar and DEET. It should be noted that the MP1 neurons have been previously implicated in shock- and sugar-reinforced learning and memory expression [6, 7, 9, 23]. Since we observed a strong requirement for MP1 neurons in behavioral DEET learning (Figures 2E and 2H), we conclude that MP1 activity is likely to represent aversive reinforcement signals to mushroom body neurons. As expected, transmission from mushroom body neurons is required for the expression of DEET memory (Figure S3).

**Figure 3:**
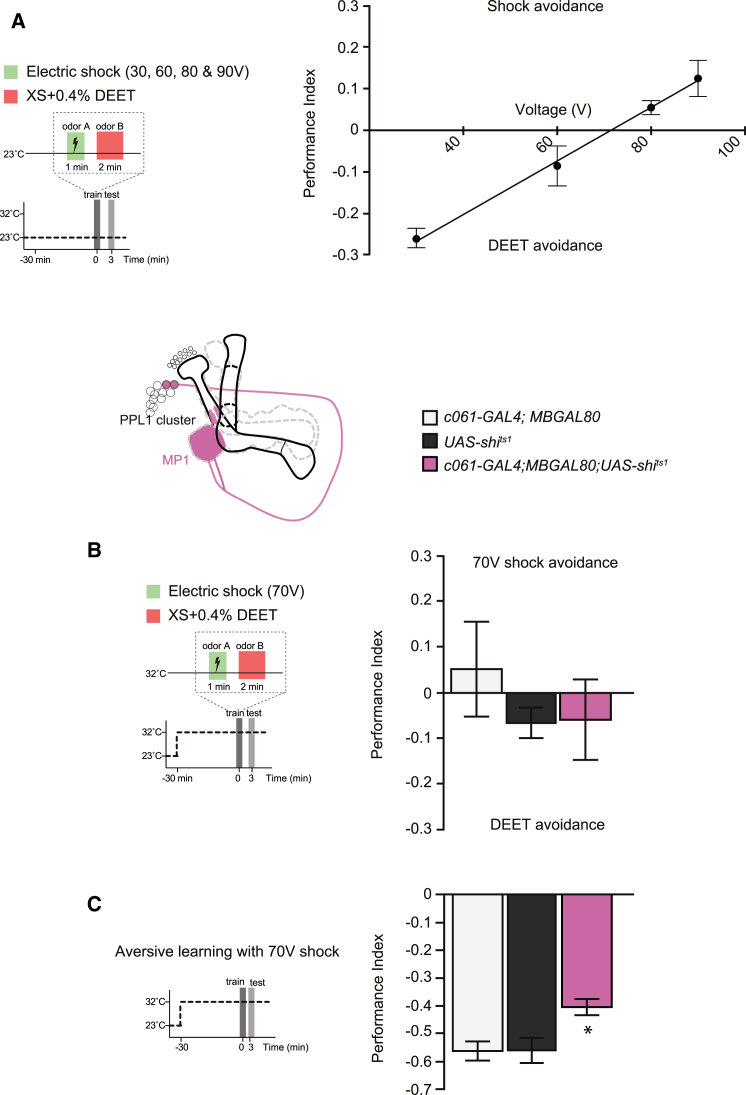
Discrete Aversive Signals Use Common Reinforcing Dopaminergic Neurons (A) Flies were differentially conditioned by pairing of one odor with electric shock of varying magnitude and the other odor with 0.4% DEET in sugar carrier. They were then immediately tested for olfactory preference. Flies avoided the odor that had been previously paired with 80 or 90 V but preferred the odor if it was paired with 30 or 60 V. Linear regression suggested that the intersecting point of equivalence between DEET and shock reinforcement was ∼70 V (R^2^ = 0.68). n ≥ 8. (B) Blockade of MP1 neurons did not alter the equivalent value of 0.4% DEET and 70 V. Flies trained with 70 V versus 0.4% DEET showed no learned odor preference. Performance of c061; MBGAL80; UAS-*shi*^ts1^ flies with blocked MP1 neurons was indistinguishable from that of control groups (p > 0.6, ANOVA). None of the groups were statistically significant from zero (p > 0.1, one-sample t test, n ≥ 5). (C) Blockade of MP1 neurons partially impaired aversive learning with 70 V. Performance of c061; MBGAL80; UAS-*shi*^ts1^ flies was statistically different from that of control groups (p < 0.05, ANOVA, n ≥ 5). Data are shown as mean ± SEM.

Finding a role in DEET learning for dopamine neurons that are also required for shock learning [6, 7] suggests a common reinforcement process, despite the different nature of the external unconditioned stimulus. We therefore designed a differential conditioning paradigm to further test this model. Flies were trained by pairing of one odor with DEET and the other odor with a varying intensity of electric shock. These experiments revealed an avoidance of the previously DEET-associated odor when countered with 30 or 60 V but an avoidance of the shock-paired odor when countered with 80 or 90 V (Figure 3A). Extrapolation of a curve fit between the tested points predicted 70 V as being equivalent to 0.4% DEET—which was subsequently confirmed in direct experiments (Figure 3B). Having established the point of reinforcer equivalence, we reasoned that if the shock and DEET reinforcement processes were common, blocking some of the responsible dopamine neurons would equally impair shock and DEET learning and therefore not alter equivalence. If, on the other hand, MP1 neurons contribute differently to DEET and shock reinforcement, we expected to see that blocking them would unevenly affect learned behavior and would skew performance toward one or the other, reflecting the imbalance. Strikingly, differential learning remained balanced in c061; MBGAL80; UAS-*shi*^ts1^ flies in which MP1 neurons were blocked. Importantly, this balanced valuation does not reflect a “zero versus zero” learning because the same c061; MBGAL80; UAS-*shi*^ts1^ flies only display a partial defect if they were trained with 70 V shock alone (Figure 3C). Therefore, these experiments support a model in which the reinforcing systems for 0.4% DEET and 70 V shock are similar, with MP1 being part of the system for both. In addition, it is notable that despite the relative magnitude of immediate memory scores (∼0.6 for 70 V shock and <0.3 for DEET) and the difference in respective memory persistence (hours for shock and minutes for DEET), the immediate learned value of these two aversive stimuli is comparable.

**Figure 4:**
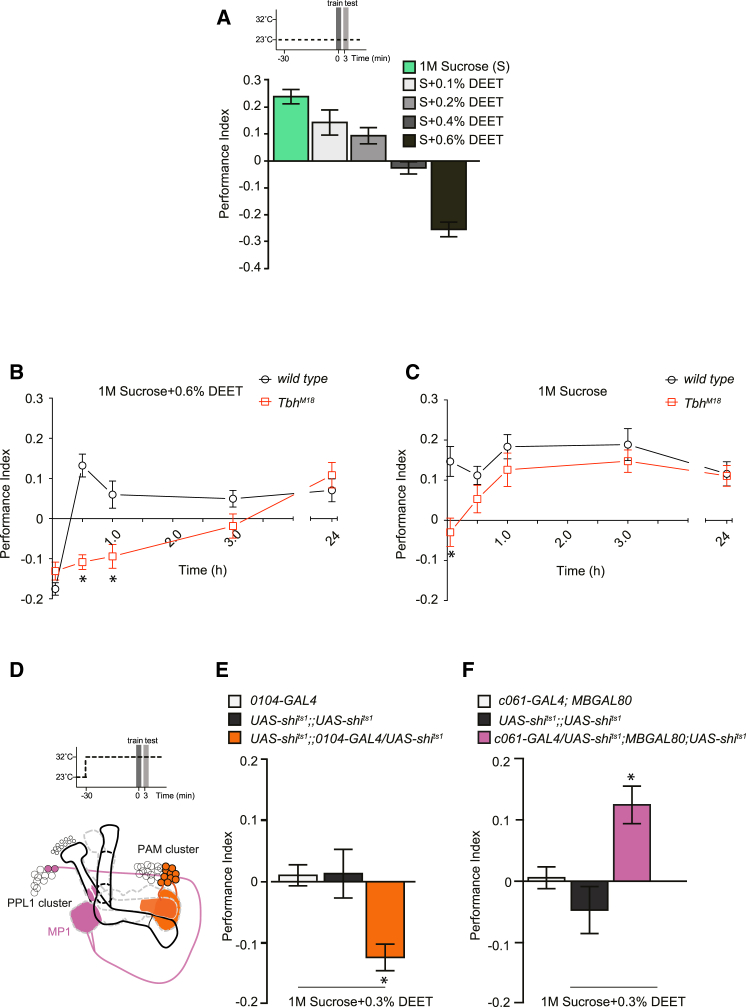
DEET:Sucrose Learning Forms Parallel Competing Appetitive and Aversive Memories (A) Learning with DEET in 1 M sucrose depends on concentration. Wild-type flies starved overnight were trained with 0% to 0.6% DEET in 1 M sucrose. Increase of DEET concentration impaired appetitive memory performance and formed robust aversive performance when presented as 0.6%. Performance with 0.6% DEET is significantly different from that of all other groups (all p < 0.0001, ANOVA, n ≥ 10). (B) Aversive DEET memory is longer lasting in *Tbh*^*M18*^ mutant flies trained with 1 M sucrose plus 0.6% DEET. Wild-type flies exhibit robust aversive memory immediately after training, but the performance converts to stable and long-lasting conditioned approach 30 min later. In contrast, *Tbh*^*M18*^ mutant flies show significant aversive memory 30 and 60 min after training, compared to that of the wild-type flies (both p < 0.005, t test). Memory performance of the *Tbh*^*M18*^ mutant flies also converts to conditioned approach by 24 hr and was not significantly different from that of wild-type flies (p > 0.1, t test). All n ≥ 10. (C) *Tbh*^*M18*^ mutant flies lack short-term sweet-taste sucrose-reinforced memory but form long-term nutrition-dependent memory. Wild-type and *Tbh*^*M18*^ mutant flies were starved overnight and trained with 1 M sucrose in 1% agar. *Tbh*^*M18*^ flies showed significantly defective immediate memory performance (p < 0.005, t test) but were indistinguishable from wild-type flies 0.5, 1, 3, and 24 hr after training (all p > 0.1, t test). All n ≥ 8. (D) Training paradigm for testing the role of MP1 and PAM dopaminergic neurons in DEET learning. The mushroom body lobe innervation of each type of dopaminergic neuron is illustrated. (E) Blockade of rewarding PAM dopaminergic neurons enhanced aversive memory performance after learning with 1 M sucrose plus 0.3% DEET. Performance of 0104-GAL4 UAS-*shi*^ts1^ flies was significantly different from that of both control groups (p < 0.005, ANOVA, n ≥ 15). (F) Blockade of the negatively reinforcing MP1 neurons enhanced appetitive memory performance after learning with 1 M sucrose plus 0.3% DEET. Performance of c061; MBGAL80; UAS-*shi*^ts1^ flies was significantly different from that of both control groups (p < 0.005, ANOVA, n ≥ 19). Data are shown as mean ± SEM.

We next investigated whether the apparent fragility of aversive DEET memory could be explained by the coformation of a more persistent sugar memory. Reasoning that these analyses would benefit from the induction of a more robust sugar memory, we first established optimal conditions for aversive memory formation with DEET-laced 1 M sucrose. Flies trained with DEET in 1 M sucrose showed a similar dose-dependent aversive learning to those trained in prior experiments with DEET in xylose and sucrose carrier, although the optimal DEET concentration for learning shifted from 0.4% to 0.6% (Figure 4A). We next tested the DEET memory performance of *Tbh*^*M18*^ mutant flies that are impaired in appetitive learning. Strikingly, whereas the behavior of wild-type flies became conditioned approach within 30 min, *Tbh*^*M18*^ flies showed a more persistent aversive memory performance, with scores remaining significantly negative 30 and 60 min after training (Figure 4B). However, the performance still converted from odor avoidance to approach by 24 hr. Since octopamine only provides short-term sweet-taste reinforcement [9], we hypothesized that persistent nutrient-dependent memory must be independently formed in *Tbh*^*M18*^ flies. Indeed, *Tbh*^*M18*^ flies trained with 1 M sucrose did not display immediate memory, but significant performance emerged 1 hr after training and remained for at least 24 hr (Figure 4C). These data support the prior model of octopamine specifically conveying short-term appetitive reinforcement and not the nutrient-dependent long-term signal [9]. In addition, they suggest that our DEET learning protocols form parallel aversive and appetitive memories. To further test a parallel memory trace model, we trained flies with 0.3% DEET and 1 M sucrose, a combination with which no immediate odor avoidance or approach performance is evident, and blocked either the rewarding or aversive dopaminergic neurons during training (Figures 4D–4F). Strikingly, blockade of the rewarding dopaminergic neurons with 0104; UAS-*shi*^ts1^ revealed significant conditioned avoidance (Figure 4E). In contrast, blockade of the negatively reinforcing MB-MP1 dopaminergic neurons with c061; MBGAL80; UAS-*shi*^ts1^ uncovered significant conditioned odor approach performance (Figure 4F). We therefore conclude that training with the compound DEET and sugar stimulus leads to the independent formation of aversive and appetitive memories. The differing stability of these competing memories subsequently determines which one of them guides learned behavior after training.

The extent to which rewarding and aversive stimuli are coded in mammalian dopaminergic neurons is hotly debated [2, 28, 29]. Recordings in the monkey have shown that some dopaminergic neurons respond to either bitter taste or an aversive air puff [30], suggesting that the quality of an aversive reinforcer may be represented. Work in flies has functionally split dopaminergic neurons into groups that are critical for reward learning and others for aversive learning [3, 5–9]. However, recent studies suggested a requirement for modulation of the aversive system in appetitive learning [9] and demonstrated a role for rewarding dopaminergic neurons in relative aversive learning [31]. In addition, imaging activity in negatively reinforcing MB-MP1 neurons revealed responses to both sweet sugar and bitter DEET. Nevertheless, the DEET reinforcement data presented here, when taken with published knowledge of shock reinforcement [7], imply that flies utilize the same, or at least an overlapping, evaluation system to convey the reinforcing effects of discrete aversive stimuli. It will be interesting to determine the respective input pathways to the negatively reinforcing dopaminergic neurons. These experiments also highlight the importance of being able to both record from and control recognizable subpopulations of dopaminergic neurons. Without intervention, it is difficult to understand whether a given dopaminergic neuron provides a reinforcement or motivational salience [2] signal.

Perhaps most surprisingly, our data demonstrate that during learning flies independently assign the value of individual components of a compound food stimulus to an odor. Rather than forming a single memory of the relative quality of the tainted sugar, they learn the bitter and sugar components in parallel. This multiplexing is further illustrated by sugars in which octopamine distinguishes between memories of sweet taste and nutrient components [9]. These results suggest that despite the integration of tastant information that occurs within the first layers of the gustatory system [15, 16, 32, 33] and provides control over food ingestion, each component also gains unprocessed access to the negative and positive arms of the reinforcement system. The fly therefore appears to retain as much information of foraging history as possible, while allowing the relative persistence of the resultant constituent memories to inform later behavior. Such a mechanism might help the fly to direct short-term foraging away from food sources that happen to be unpalatable but remember that they are usually nutritious.

## Experimental Procedures

Details of all experiments are provided in the Supplemental Experimental Procedures.

### Fly Strains

Flies were raised on cornmeal food at 25°C and 40%–50% relative humidity. The wild-type Canton-S [23], c061; MBGAL80 [23], *Tbh*^M18^ [20], *dumb*^1^ [21], UAS-*shi*^ts1^ [22], R73F07-GAL4 [24], NP1528-GAL4 [6], NP5272-GAL4 [6], UAS-*dTrpA1* [19], *Gr66a*-GAL4 [34], UAS-*hid*:UAS-rpr [17], UAS-*GCaMP3* [26], *TH*-GAL4 [27], and UAS-*IR40a*^RNAi^ [12] flies have all been described.

### Behavioral Analysis

Mixed-sex populations of 6- to 9-day-old flies were tested together in all experiments. For DEET training, groups of ∼100 flies were food deprived for 22–28 hr in vials containing 2–3 ml 1% agar and filter paper. Liquid DEET was diluted to the appropriate final concentration with a given sugar solution in 1% molten agar (in water) and shaken to create a suspension and spread onto filter paper. Dried papers were rolled into training tubes. Training paradigms are indicated in the figure legends.

A performance index (PI) was calculated as the number of flies approaching (appetitive) or avoiding (aversive) the conditioned odor minus the number going the other direction, divided by the total number of flies in the experiment. A single PI is the average of two experiments in which identical genotype flies are trained with the reciprocal reinforced/non-reinforced odor combination. Odors were 3-octanol and 4-methylcyclohexanol.

### Two-Photon In Vivo Calcium Imaging

Adult, food-deprived UAS-GCaMP3; THGAL4 flies were waxed to a custom-built imaging chamber, and the head capsule was removed under ice-cold sugar-free saline. Two-photon imaging was performed with a custom-made imaging setup as described [35].

### Statistical Analysis

Statistical analyses were performed with PRISM (GraphPad Software) and are specifically referenced in the figure legends.

## Supplemental Information

Document S1. Supplemental Experimental Procedures and Figures S1–S3

Document S2. Article plus Supplemental Information
