# Supplementary material for: Drosophila Learn Opposing Components of a Compound Food Stimulus
Source: Curr Biol. 2014 Aug 4;24(15):1723–30. doi: 10.1016/j.cub.2014.05.078 (PMC4131107; doi:10.1016/j.cub.2014.05.078)
Supplement: Document S1. Supplemental Experimental Procedures and Figures S1–S3 [file mmc1.pdf]

**Current Biology, Volume *24***

**Supplemental Information**

# *Drosophila* Learn Opposing Components of a Compound Food Stimulus

**Gaurav Das, Martín Klappenbach, Eleftheria Vrontou, Emmanuel Perisse, Christopher M. Clark, Christopher J. Burke, and Scott Waddell**

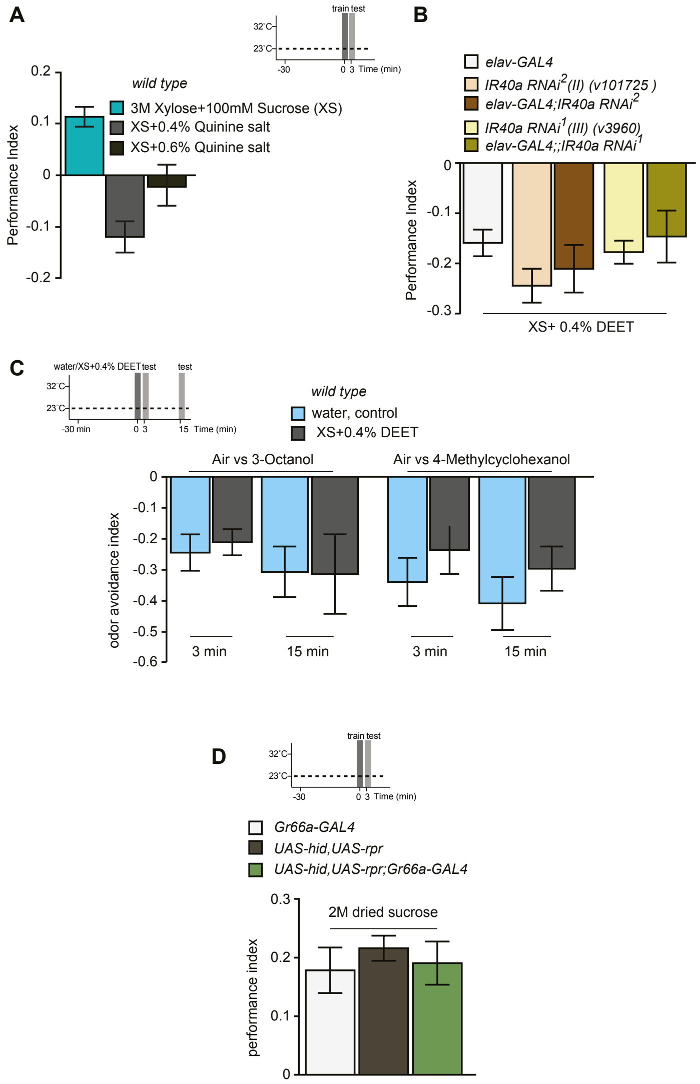


**Figure S1. Aversive learning with bitter quinine and requirements for DEET learning. Related to Figure 1.**

(A) Aversive learning with bitter quinine hydrochloride salt shows similar concentration dependence to learning with DEET. Wild type flies starved overnight were trained with 0.4 and 0.6% quinine salt in carrier sugars and were immediately tested for olfactory memory. Robust aversive memory was formed with 0.4% quinine but performance was negligible with 0.6%. n≥ 6.

(B) Learning with 0.4% DEET in sugar carrier is independent of the IR40a olfactory DEET receptor. Flies expressing either the v101725 or v3960 *IR40a* UAS-RNAi construct driven by *elav-GAL4* were trained with 0.4% DEET in sugar carrier. No significant difference in DEET learning was observed compared to control flies. (p>0.4, n≥ 3-6).

(C) DEET feeding does not alter olfactory acuity. Wild type flies fed 0.4% DEET in carrier or water for 2 min were tested immediately, or 15 min afterwards. No significant difference was observed between the relevant experimental and control groups, (all p>0.5, n≥ 4-6).

(D) Flies with ablated *Gr66a*-GAL4 bitter-sensing neurons are not impaired for sugar learning. No statistical differences were apparent between ablated flies and control groups (p>0.7, n≥ 4-6).

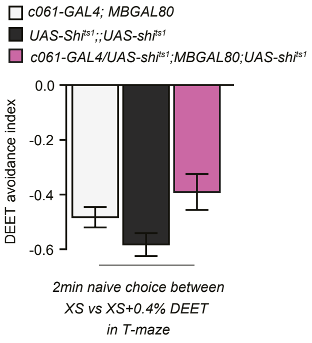


**Figure S2. MP1 neurons are not required for DEET avoidance in naïve flies. Related to Figure 2.**
Blocking the MP1 neurons with c061; MBGAL80; UAS-*shi*$^{ts1}$ did not significantly impair naïve DEET avoidance. (p>0.3, except with UAS-*shi*$^{ts1}$ p=0.03, ANOVA, n= 8).

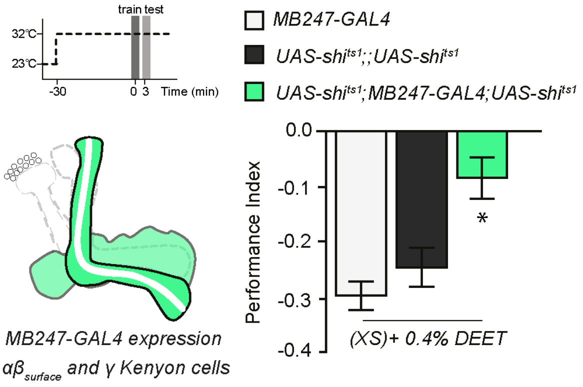


**Figure S3. DEET memory performance requires mushroom body output. Related to Figure 2.**
Blocking transmission from αβ surface and γ mushroom body neurons with MB247-GAL4 driven UAS-*shi*$^{ts1}$ (X, III) significantly impaired DEET learning ($p < 0.005$, ANOVA, n ≥ 12). Schematic depicts the experimental protocol and MB247-GAL4 expression pattern (green).

## Supplemental Experimental Procedures

### Fly strains

Fly stocks were raised on standard cornmeal food at 25°C and 40-50% relative humidity. The wild-type *Drosophila* strain used in this study is Canton-S. The MB-MP1 expressing c061;MBGAL80 flies are described. MBGAL80 suppresses GAL4 activity in mushroom body neurons in these flies [23]. *Tbh*$^{M18}$ and *dumb*$^{1}$ mutant flies are described [20,21]. We used flies carrying UAS-*shi*$^{ts1}$ on the third chromosome [UAS-*shi*$^{ts1}$ (III)] or on the X and third chromosome [UAS-*shi*$^{ts1}$ (X, III)] [22]. Flies expressing *shi*$^{ts1}$ in MB-MP1 neurons were generated by crossing UAS-*shi*$^{ts1}$ (X, III) (Fig. 2E) or UAS-*shi*$^{ts1}$ (III) males (Fig. 2H, 3B and C) to homozygous c061;MBGAL80 females. Flies expressing *shi*$^{ts1}$ in MB-MV1 or M3 neurons were generated by crossing UAS-*shi*$^{ts1}$ (X and III) females to homozygous R73F07-GAL4 males [24] or NP1528-GAL4 and NP5272-GAL4 [6] males, respectively. MB247-GAL4 [S1] homozygous males were crossed to UAS-*shi*$^{ts1}$ (X and III) females to drive *shi*$^{ts1}$ expression in mushroom body neurons. Heterozygous control flies were generated by crossing UAS-*shi*$^{ts1}$ (III or X and III) or GAL4 flies to wild-type flies. Flies expressing dTrpA1 in bitter taste neurons were established by crossing *UAS-dTrpA1* [19] females to homozygous *Gr66a*-GAL4 males [35]. To ablate Gr66a neurons, UAS-*hid*:UAS-rpr [17] flies were crossed to *Gr66a*-GAL4. To generate flies expressing *dTrpA1* in bitter taste neuron of *dumb*$^{1}$ flies, *UAS-dTrpA1; dumb*$^{1}$ females were crossed to *Gr66a*-GAL4; *dumb*$^{1}$ males. UAS-*GCaMP3* flies are described [26] and were crossed to *TH*-GAL4 flies [27] for *in vivo* Calcium imaging. We knocked down *IR40a* using UAS-RNAi lines [12] driven pan-neurally by *elav*-GAL4 (X).

### Behavioral analysis

Mixed sex populations of 6-9 day old flies raised at 25°C were tested together in all behavior experiments. Prior to training flies with DEET, groups of ~100 flies were food-deprived for 22-28 h in vials containing 2-3 ml 1% agar and a strip of filter paper. For CS+ reinforcement, liquid DEET was diluted to a final concentration of 0.4% (or as otherwise stated) along with 3M Xylose and 100mM Sucrose, in 1% molten agar (in water), shaken well to create a suspension and ~3ml was rapidly spread in an even layer on a 50 X 70 mm piece of filter paper, backed with Parafilm. 1% agar in water was used for the CS-. After drying for 30-40 min, the papers were rolled into T-maze training tubes. For experiments at 32°C, the training papers were further dried for ~30 minutes at 32°C. For both 23°C and 32°C experiments, humidity was maintained below 55%.

Flies were trained by first exposing them to one odor with 1% agar for 2 min, then clear airflow for 30 s. They were then tapped into a training tube, lined with carrier sugar and DEET and exposed to a second odor for 2 min. To test 3 min memory, flies were immediately transferred to the T-maze and given 2 min to choose between the two odors (Figs 1A, C, D, 2A, E-H). For 15, 30  60 min, 3 h and 24 h memory testing, flies were transferred back to starvation tubes before being reloaded into the T-maze for testing (Fig 1D, 4B-C). For all conditioning experiments, Performance Index (PI) was calculated as the number of flies approaching (appetitive memory) or avoiding (aversive memory) the conditioned odor minus the number of flies going the other direction, divided by the total number of flies in the experiment. A single PI value is the average score from flies of the identical genotype tested with the reciprocal reinforced/non-reinforced odor combination. Odors used were 3-octanol (OCT, 7 µl in 8 ml mineral oil) and 4-methylcyclohexanol (MCH, 6-7 µl in 8 ml mineral oil).

To block specific neurons with UAS-*shi*$^{ts1}$, flies in vials were transferred to 32°C, according to the schematics accompanying each figure (Figs 2D 3B,C and 4D).

For memory implantation experiments using UAS-*dTrpA1* mediated neural

activation (Figs 1F and 2C) 8-11 day old flies raised at room temperature and starved overnight were presented with one odor at restrictive 32°C for 2 min. During the next 45 s, they were transferred into a training tube with the other odor at permissive 23°C and left for 2 min. Flies were tested for immediate memory, or returned to starvation vials for testing at 3 h.

Aversive shock memory (Fig 3C) was assayed as described [31,S2]. Before training groups of ~100 flies were housed for 18–20 h in a 25 ml vial containing standard cornmeal/agar food and a piece of filter paper. Reinforcement was 70V.

For differential aversive experiments (Fig 3A and B), flies were prepared as above for aversive memory and were conditioned as follows; 1 min one odor with twelve 30, 60, 70 or 90 V shocks at 5 s inter-stimulus interval (ISI), 45 s fresh air and followed by 2 min second odor with 0.4% DEET in carrier. They were immediately tested for memory performance.

For testing odor-avoidance post DEET feeding (Fig. S1C), flies were allowed 2 min to feed on 0.4% DEET in sugar carrier (3M xylose+100mM sucrose) and 1% agar, or just 1% agar inside T-maze training tubes. They were subsequently tested immediately or after 15 min for their preference between OCT/MCH and odor free airflow. An odor avoidance index was calculated, similar to PI value described above. Naïve DEET avoidance (Fig S2A) was assay in the T-maze by allowing flies 2 min to choose between a tube lined with sugar carrier or sugar carrier with DEET in 1% agar. A DEET avoidance index was calculated as for PI value.

**Feeding assay**

Feeding was measured as described [14,S3] with modifications. Sugar carrier (3M xylose +100mM sucrose) + varying concentration of DEET mixture was prepared in 1% agar with 0.4% FD&C Blue No. 1 dye. Flies were given 5 min to feed on the respective mixture accompanied by MCH/OCT odor flow and were then immediately frozen. 50 flies were then processed to release and isolate the consumed blue dye and absorbance was measured at 625 nm.

**2-Photon *in vivo* calcium imaging**

Adult, food deprived UAS-GCaMP3;THGAL4 flies were waxed to a custom built imaging chamber and the head capsule was removed under ice-cold sugar free saline (108 mM NaCl, 5 mM KCl, 8.2 mM $MgCl_2$, 4 mM $NaHCO_3$, 1 mM $NaH_2PO_4$, 15 mM Ribose, 5 mM HEPES, pH 7.5). Two-photon imaging was performed using a custom made imaging set-up as described [38]. In brief, fluorescence was excited using 140 fs pulses centered on 910 nm generated by a Ti-sapphire laser (Chameleon Ultra II, Coherent), attenuated by a Pockels cell (Conoptics 302RM). Brains were imaged using a Movable Objective Microscope (Sutter) with a Zeiss 203, 1.0 NA W-Plan-Apochromat objective. The microscope was controlled using MPScope 2.0 [S4] via a PCI-6110 DAQ board (National Instruments). Images were acquired at approximately 4 Hz. 0.4% DEET + 1M sucrose was delivered to the fly manually through a tubing while the fly was observed using a Stingray CCD camera (Allied Vision Technologies). Images were converted to Analyze format and motion corrected, when needed, by maximizing the pixel-by-pixel correlation between each frame and a reference frame. DF/F traces were calculated in MATLAB using manually drawn regions of interest (ROIs) for the background and brain structure of interest. Activity maps were generated from Gaussian-smoothed, background subtracted images. A baseline fluorescence image was calculated as the average over a 10 s prestimulus interval. Final traces were generated in Prism 6 (GraphPad Software).

## Statistical Analysis

All statistical analyses were performed using PRISM (GraphPad Software) and tests are specifically referenced in the figure legends. For all T-maze behavioral experiments data is represented as mean ± standard error of the mean (SEM). Analysis of variance (ANOVA) was followed by planned pairwise comparisons between the relevant groups with a Tukey honestly significant difference HSD post hoc test. Listed p values are multiplicity adjusted. To determine whether values were significantly different form zero, one sample t-tests were performed. Linear regression was performed for Figure 3A (Y=0.006428*X-0.4592). In Figure 4B and C, multiple t-test was performed between the two groups at each time point, with Holm-Sidak correction for multiple comparisons.

## Supplemental References

S1. Zars, T., Fischer, M., Schulz, R., and Heisenberg, M. (2000). Localization of a short-term memory in Drosophila. Science *288*, 672–675.

S2. Tully, T., and Quinn, W.G. (1985). Classical conditioning and retention in normal and mutant Drosophila melanogaster. J Comp Physiol [A]. *157*, 263-277.

S3. Tanimura, T., Isono, K., Takamura, T., and Shimada, I. (1982). Genetic dimorphism in the taste sensitivity to trehalose in Drosophila melanogaster. J. Comp. Physiol. A Neuroethol. Sens. Neural Behav. Physiol. *147*, 433-437.

S4. Nguyen, Q.T., Tsai, P.S., and Kleinfeld, D. (2006). MPScope: a versatile software suite for multiphoton microscopy. J. Neurosci. Methods. *156*, 351-359.
