# Supplementary material for: Drosophila Learn Opposing Components of a Compound Food Stimulus
Source: Curr Biol. 2014 Aug 4;24(15):1723–30. doi: 10.1016/j.cub.2014.05.078 (PMC4131107; doi:10.1016/j.cub.2014.05.078)
Supplement: Document S2. Article plus Supplemental Information [file mmc2.pdf]

Current Biology 24, 1723–1730, August 4, 2014 ©2014 The Authors http://dx.doi.org/10.1016/j.cub.2014.05.078

Report

Drosophila Learn Opposing Components of a Compound Food Stimulus

Gaurav Das,[1] Martín Klappenbach,[2] Eleftheria Vrontou,[1] Emmanuel Perisse,[1] Christopher M. Clark,[3] Christopher J. Burke,[3] and Scott Waddell[1,3,*]
[1]Centre for Neural Circuits and Behaviour, University of Oxford, Tinsley Building, Mansfield Road, Oxford OX1 3SR, UK
[2]Laboratorio de Neurobiología de la Memoria, Facultad de Ciencias Exactas y Naturales, IFIBYNE-CONICET, Universidad de Buenos Aires, Buenos Aires C1428EGA, Argentina
[3]Department of Neurobiology, University of Massachusetts Medical School, 364 Plantation Street, Worcester, MA 01605, USA

Summary

Dopaminergic neurons provide value signals in mammals and insects [1–3]. During *Drosophila* olfactory learning, distinct subsets of dopaminergic neurons appear to assign either positive or negative value to odor representations in mushroom body neurons [4–9]. However, it is not known how flies evaluate substances that have mixed valence. Here we show that flies form short-lived aversive olfactory memories when trained with odors and sugars that are contaminated with the common insect repellent DEET. This DEET-aversive learning required the MB-MP1 dopaminergic neurons that are also required for shock learning [7]. Moreover, differential conditioning with DEET versus shock suggests that formation of these distinct aversive olfactory memories relies on a common negatively reinforcing dopaminergic mechanism. Surprisingly, as time passed after training, the behavior of DEET-sugar-trained flies reversed from conditioned odor avoidance into odor approach. In addition, flies that were compromised for reward learning exhibited a more robust and longer-lived aversive-DEET memory. These data demonstrate that flies independently process the DEET and sugar components to form parallel aversive and appetitive olfactory memories, with distinct kinetics, that compete to guide learned behavior.

Results and Discussion

DEET has been reported to drive aversive behavior in flies through olfactory [10–12] and gustatory [13] pathways. We therefore used a low concentration presented in solid medium (1% agar) to decrease the effects of volatile DEET and increase the chance that flies would taste and perhaps ingest it. To further encourage flies to sample DEET, we increased its palatability by adding it to a mixture of sweet sugars—3 M xylose and 100 mM sucrose (from here on referred to as "carrier"). Xylose is detected by sweet-sensitive gustatory neurons and is palatable to flies, but it contributes no measurable nutrient value [14]. The low concentration of sweet and nutritious sucrose was added to further increase palatability [14]. We first determined the optimum DEET concentration by adding increasing amounts to sugar carrier and conditioning hungry flies by pairing the exposure of the second of two odors with DEET presentation.

Flies trained with only the sugar carrier showed a significant appetitive memory (Figure 1A). In contrast, those trained with increasing amounts of DEET formed aversive memory, with the score rising in line with the increase in DEET concentration, up to 0.4%. Surprisingly, flies trained with 0.8% DEET did not exhibit significantly negative aversive memory scores, suggesting a change in the flies' perception of DEET at this concentration. We therefore tested the effect of 0.4% and 0.8% DEET on fly feeding by measuring ingestion marked with blue food dye (Figure 1B). Whereas flies ate significant amounts of food containing sugar carrier, both 0.4% and 0.8% DEET strongly suppressed feeding behavior. However, whereas flies ate a measurable amount of dye with 0.4% DEET, ingestion was abolished with 0.8% DEET. These data suggest that the failure to train flies with 0.8% DEET reflects an inhibition of sampling by the proboscis and perhaps ingestion of DEET and sugar. To further test a requirement for feeding in learning, we attempted to train flies that were not hungry or with 0.4% DEET without sugar carrier (Figure 1C). Both of these conditions significantly impaired aversive learning when compared to hungry flies trained with 0.4% DEET in sugar carrier. We also observed a similar concentration-dependent aversive memory formation when flies were trained with bitter-tasting quinine that was mixed with sugar carrier (Figure S1A available online). Furthermore, flies that were defective in the IR40a olfactory route of DEET detection displayed normal DEET learning (Figure S1B). We therefore conclude that robust learning with 0.4% DEET-laced sugar requires the flies to attempt to eat DEET and that low DEET concentrations convert the conditioned approach that is formed when flies are trained with the sugar carrier into a conditioned aversion.

We next measured the persistence of DEET memory by conditioning flies and testing their odor preference at extended times after training (Figure 1D). Whereas aversive memory performance was robust immediately after training, no statistically significant performance was evident 15 min later. Aversive memory formed with 0.4% DEET is therefore surprisingly labile. DEET and quinine can be sensed by bitter-taste neurons [13, 15, 16], and ablation of bitter-sensing neurons with *Gr66a*-GAL4-directed expression of cell-death genes [17] partially impaired DEET, but not sugar, learning (Figures 1E and S1D). We therefore tested whether flies could be aversively conditioned by pairing odor presentation with artificial bitter-taste neuron [16, 18] activation, achieved by expression of UAS-*dTrpA1* (Figure 1F). The dTrpA1 gene encodes a transient receptor potential (TRP) channel that conducts Ca^{2+} and depolarizes neurons when flies are exposed to temperature >25°C [19]. *Gr66a*-GAL4, UAS-*dTrpA1*, and *Gr66a*-GAL4; UAS-*dTrpA1* flies were conditioned by presentation of the first odor with activating 32°C and were immediately tested for memory. *Gr66a*-GAL4; UAS-*dTrpA1* flies exhibited aversive memory that was statistically different from that of all other groups (Figure 1F). However, unlike flies conditioned with DEET (Figure 1D), significant memory remained 3 hr after training (Figure 1F). The differing persistence could result from artificial stimulation of bitter neurons being stronger than DEET activation, in addition to lacking plausible competition from a copresented sugar stimulus.

*Correspondence: scott.waddell@cncb.ox.ac.uk
This is an open access article under the CC BY license (http://creativecommons.org/licenses/by/3.0/).

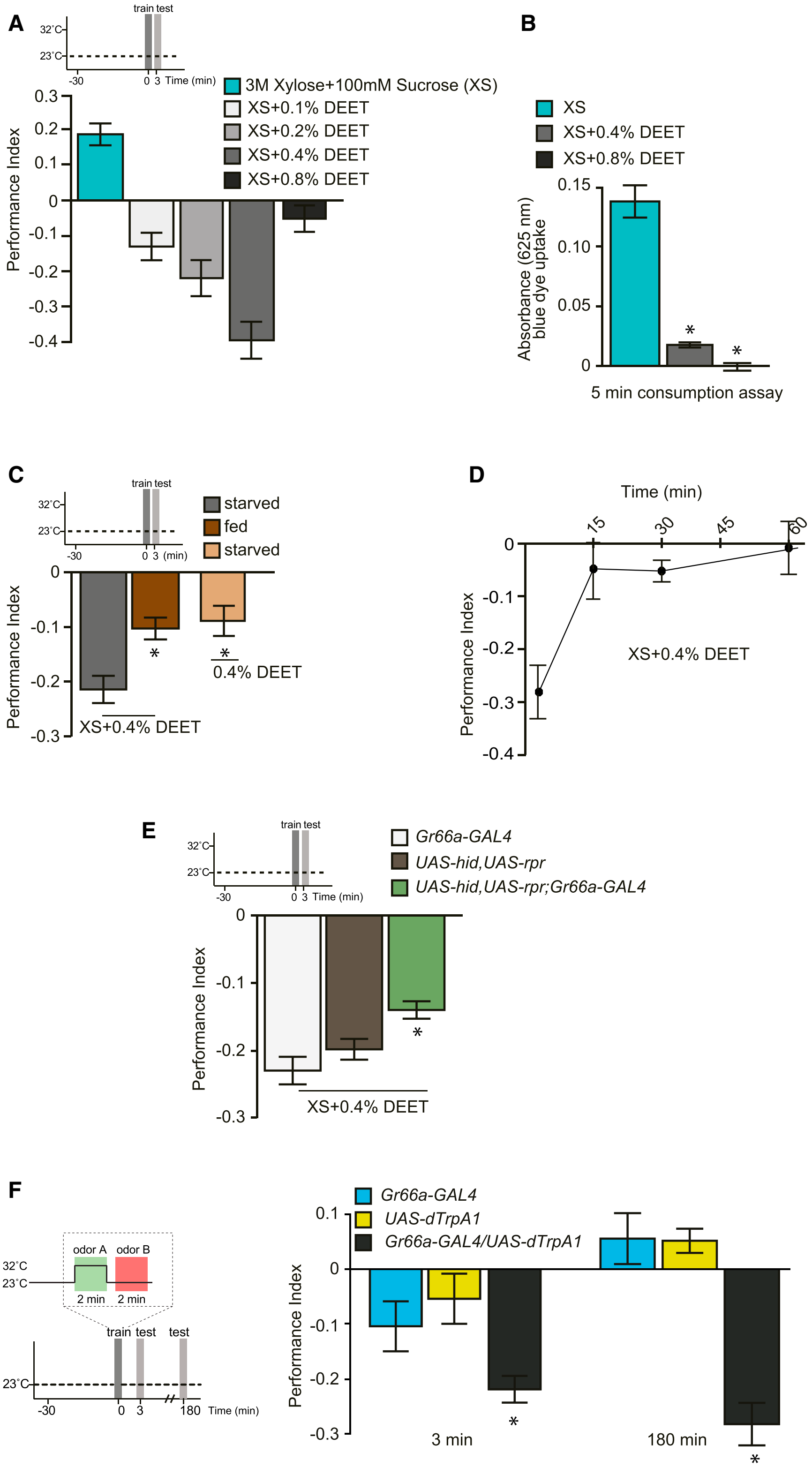

Figure 1. Aversive Olfactory Learning with DEET Reinforcement

(A) Learning with DEET depends on concentration. Wild-type flies starved overnight were trained with 0%–0.8% DEET with a sugar carrier (3 M xylose plus 100 mM sucrose). Aversive memory performance increased with DEET concentrations up to 0.4% but was negligible with 0.8% DEET. Learning with 0.4% DEET is significantly different from that with both 0.1% and 0.8% DEET (both $p < 0.001$). All other group wise comparisons are not significant (all $p > 0.05$). ANOVA followed by Tukey's multiple comparison test was performed. p values are multiplicity adjusted (ANOVA). $n \geq 5$.
(B) DEET inhibits ingestion. The amount of sugar carrier plus DEET (0.4% or 0.8%) ingested in 5 min was quantified using FD&C Blue No. 1 dye supplementation. Flies consumed sugar carrier alone, but inclusion of 0.4% or 0.8% DEET significantly reduced consumption. However, flies consumed statistically significant amounts of dye presented with 0.4% DEET, but not with 0.8% DEET ($p = 0.002$ and $p = 0.8427$, respectively, from zero; one-sample t test, $n \geq 5$).
(C) DEET learning is most robust when flies ingest. Hungry flies display robust immediate aversive memory with 0.4% DEET presented with sugar carrier. However, both satiated flies and those trained with 0.4% DEET without sugar carrier exhibited significantly less aversive memory performance (both $p < 0.05$, ANOVA, $n \geq 10$).
(D) DEET memory is labile. DEET reinforced memory decayed rapidly and was not significant 15 min after training ($p < 0.05$ versus 3 min performance and $p > 0.9$ versus 30 and 60 min, ANOVA, $n \geq 6$).
(E) Ablation of bitter-taste neurons impairs DEET learning. Flies expressing UAS-*hid* and UAS-*rpr* in *Gr66a*-GAL4 cells were trained with 0.4% DEET in sugar carrier (3 M xylose and 100 mM sucrose) and were immediately tested for memory performance. The performance of these flies was statistically different than that of the control groups ($p < 0.05$, ANOVA, $n \geq 23$).
(F) Aversive memory can be implanted with bitter-taste neuron activation contingent with odor presentation. Flies were trained by pairing dTrpA1-mediated activation of *Gr66a*-GAL4 bitter gustatory neurons with odor as shown in the schematic. Significant aversive memory was formed in *Gr66a*-GAL4/UAS-*dTrpA1* flies that persisted for at least 3 hr (at both time points $p < 0.005$, ANOVA, $n \geq 8$).
Data are shown as mean ± SEM. Asterisks denote significant difference between marked group and the relevant controls. See also Figure S1.

Octopamine is required to convey the reinforcing effects of sweet taste [9]. We therefore tested DEET learning in Tbh^{M18} mutant flies that cannot synthesize octopamine [20] (Figure 2A). Whereas appetitive conditioning with 1 M sucrose was significantly impaired in Tbh^{M18} flies, aversive learning with 0.4% DEET was indistinguishable from that of wild-type flies. Therefore, octopamine is not required for DEET learning.

Electric-shock-reinforced aversive memory formation also requires specific dopaminergic neurons and the DopR1 dopamine receptor [5–7, 21]. We therefore first determined whether DEET learning required the DopR1 receptor (Figure 2B). Mutant $dumb^1$ flies that are defective for the DopR1 dopamine receptor did not display aversive learning with DEET. Similarly, aversive learning with artificial activation of bitter-taste neurons was abolished in $dumb^1$ flies (Figure 2C).

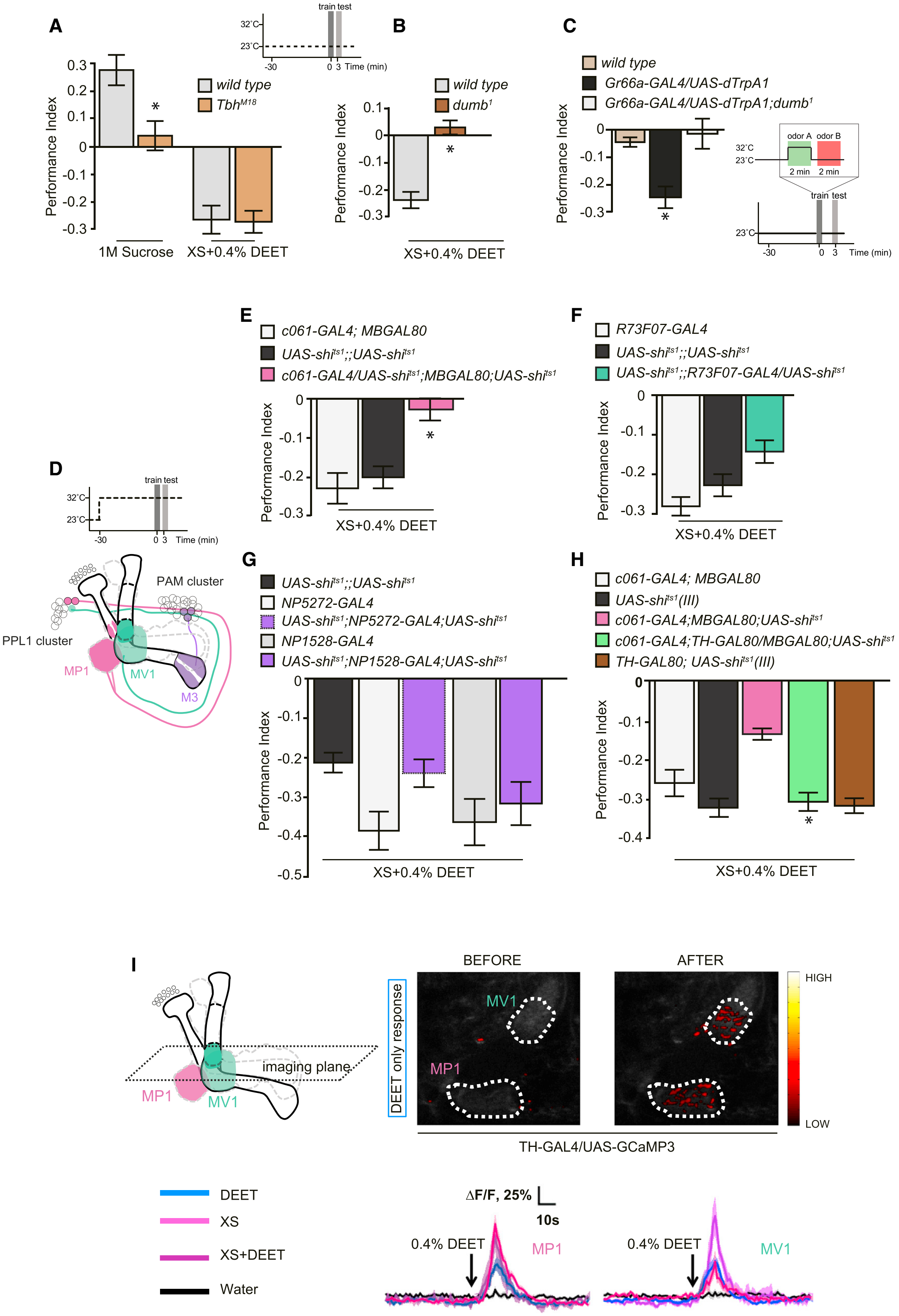

Figure 2. DEET Reinforcement Involves Specific Dopaminergic Neurons

(A) DEET learning does not require octopamine. Appetitive memory formation with 1 M sucrose was significantly impaired in *Tbh*^{M18} flies ($p < 0.05$, t test, $n \geq 4$), whereas aversive memory with DEET was statistically indistinguishable from that of control flies ($p > 0.5$, t test, $n \geq 6$).

(B) DEET memory formation was significantly impaired in *dumb*^{1} mutant flies ($p < 0.001$, t test, $n \geq 10$).

(legend continued on next page)

The MB-MP1, MB-MV1, and MB-M3 classes of dopamine neuron have been previously implicated in shock learning [6, 7] (Figure 2D). To test whether either of these neurons were required for DEET learning, we expressed the dominant temperature-sensitive UAS-*shibire*^{ts1} transgene [22] in MP1, MV1, and M3 neurons using the c061; MBGAL80, R73F07, and NP5272 and NP1528 GAL4 drivers [7, 23, 24], respectively. The *shi*^{ts1} transgene permitted blockade of the respective neurons by performing DEET conditioning experiments at the restrictive temperature of 31°C. This analysis revealed significantly impaired DEET learning performance when MP1 neurons were blocked (Figure 2E) but nonsignificant effects when either MV1 (Figure 2F) or M3 (Figure 2G) neurons were compromised. Blockade of MP1 neurons, however, did not significantly affect DEET avoidance in naive flies (Figure S2). To further support a role for the dopaminergic MP1 neurons in c061; MBGAL80, we removed them from the expression pattern by including a *TH*-GAL80 transgene [25]. When the remaining cells were blocked during conditioning, flies exhibited levels of DEET learning that were indistinguishable from those of wild-type flies (Figure 2H). We therefore conclude that MP1 neurons are critical for DEET learning, whereas MV1 and M3 neurons contribute a lesser role. We note that prior work implicated the MV1 and M3 neurons in the formation of more persistent forms of shock-reinforced aversive memory [6, 7].

We next used live imaging to determine whether DEET ingestion activated the MP1 dopamine neurons. We expressed UAS-*GCaMP3* [26] in dopaminergic neurons with *TH*-GAL4 [27] and imaged DEET-evoked changes in fluorescence in the dopaminergic neuron processes on the mushroom body (Figure 2I). These analyses revealed strong activation of the MP1 innervated heel and MV1 innervated junction regions of the mushroom body while presenting flies with both 0.4% DEET in sugar carrier, sugar carrier alone, and DEET alone. In comparison, water presentation did not activate the MP1 and MV1 neurons. Therefore, functional imaging does not reveal obvious valence specificity of MP1 and MV1 signals, being activated by both sugar and DEET. It should be noted that the MP1 neurons have been previously implicated in shock- and sugar-reinforced learning and memory expression [6, 7, 9, 23]. Since we observed a strong requirement for MP1 neurons in behavioral DEET learning (Figures 2E and 2H), we conclude that MP1 activity is likely to represent aversive reinforcement signals to mushroom body neurons. As expected, transmission from mushroom body neurons is required for the expression of DEET memory (Figure S3).

Finding a role in DEET learning for dopamine neurons that are also required for shock learning [6, 7] suggests a common reinforcement process, despite the different nature of the external unconditioned stimulus. We therefore designed a differential conditioning paradigm to further test this model. Flies were trained by pairing of one odor with DEET and the other odor with a varying intensity of electric shock. These experiments revealed an avoidance of the previously DEET-associated odor when countered with 30 or 60 V but an avoidance of the shock-paired odor when countered with 80 or 90 V (Figure 3A). Extrapolation of a curve fit between the tested points predicted 70 V as being equivalent to 0.4% DEET—which was subsequently confirmed in direct experiments (Figure 3B). Having established the point of reinforcer equivalence, we reasoned that if the shock and DEET reinforcement processes were common, blocking some of the responsible dopamine neurons would equally impair shock and DEET learning and therefore not alter equivalence. If, on the other hand, MP1 neurons contribute differently to DEET and shock reinforcement, we expected to see that blocking them would unevenly affect learned behavior and would skew performance toward one or the other, reflecting the imbalance. Strikingly, differential learning remained balanced in c061; MBGAL80; UAS-*shi*^{ts1} flies in which MP1 neurons were blocked. Importantly, this balanced valuation does not reflect a “zero versus zero” learning because the same c061; MBGAL80; UAS-*shi*^{ts1} flies only display a partial defect if they were trained with 70 V shock alone (Figure 3C). Therefore, these experiments support a model in which the reinforcing systems for 0.4% DEET and 70 V shock are similar, with MP1 being part of the system for both. In addition, it is notable that despite the relative magnitude of immediate memory scores (~0.6 for 70 V shock and <0.3 for DEET) and the difference in respective memory persistence (hours for shock and minutes for DEET), the immediate learned value of these two aversive stimuli is comparable.

We next investigated whether the apparent fragility of aversive DEET memory could be explained by the coformation of a more persistent sugar memory. Reasoning that these analyses would benefit from the induction of a more robust sugar memory, we first established optimal conditions for aversive memory formation with DEET-laced 1 M sucrose. Flies trained with

(C) Aversive memory formation with bitter-taste neuron activation is impaired in *dumb*^{1} mutant flies. Performance of *Gr66a*-GAL4/UAS-*dTrpA1* flies was statistically different from that of *Gr66a*-GAL4/UAS-*dTrpA1*; *dumb*^{1} and wild-type flies (both $p < 0.005$, ANOVA, $n \geq 11$).

(D) Schematic of the training paradigm for testing the role of the specific MP1, MV1, and M3 dopaminergic neurons in DEET learning. The innervation zone of each type of dopaminergic neuron on the ipsilateral mushroom body lobe is illustrated. MP1 and MV1 neuron cell bodies reside in the PPL1 cluster, whereas M3 is in the PAM cluster.

(E) Blockade of the MP1 neurons with c061; MBGAL80; UAS-*shi*^{ts1} significantly impaired DEET learning ($p < 0.001$, ANOVA, $n \geq 12$).

(F) Blockade of the MV1 neurons with R73F07; UAS-*shi*^{ts1} did not significantly impair DEET learning ($p < 0.05$ versus R73F07, but $p > 0.05$ versus UAS-*shi*^{ts1}, ANOVA, $n \geq 20$).

(G) Blockade of the M3 neurons with NP5272; UAS-*shi*^{ts1} or NP1528; UAS-*shi*^{ts1} did not significantly impair DEET learning ($p > 0.05$, ANOVA, $n \geq 7$).

(H) Removal of *shi*^{ts1} transgene expression from dopaminergic neurons in c061-GAL4; MBGAL80; UAS-*shi*^{ts1} (III) flies, by inclusion of *TH*-GAL80, significant restores DEET learning ($p < 0.001$ versus c061-GAL4; MBGAL80; UAS-*shi*^{ts1} flies). Further, performance of c061-GAL4; *TH*-GAL80/MB-GAL80; UAS-*shi*^{ts1} flies was indistinguishable from that of other control groups (all $p > 0.5$, ANOVA, $n \geq 8$).

(I) Feeding of 0.4% DEET in water evokes an increase in intracellular Ca^{2+} in MP1 and MV1 neurons, measured using UAS-*GCaMP3* expression driven by *TH*-GAL4. A time course of DEET-evoked GCaMP3 responses ($\Delta F/F$) in MP1 and MV1 processes in the mushroom body lobes, measured at the indicated regions of interest (dashed outlines), is shown. Averaged traces are shown as solid lines, and shaded areas represent the SEM. The arrow indicates the onset of DEET presentation. Inset panels show pseudocolored activity maps of neural responses before and after DEET presentation, overlaid on grayscale images of baseline fluorescence. Additionally, DEET in carrier (3 M xylose and 100 mM sucrose) and carrier alone evoke a significant increase in intracellular Ca^{2+} in MP1 and MV1 neurons. Water presentation produced a negligible response. Average traces (from seven to 17 repetitions in three flies per condition) are shown for all groups, except 0.4% DEET by itself.

Data are shown as mean ± SEM. See also Figures S2 and S3.

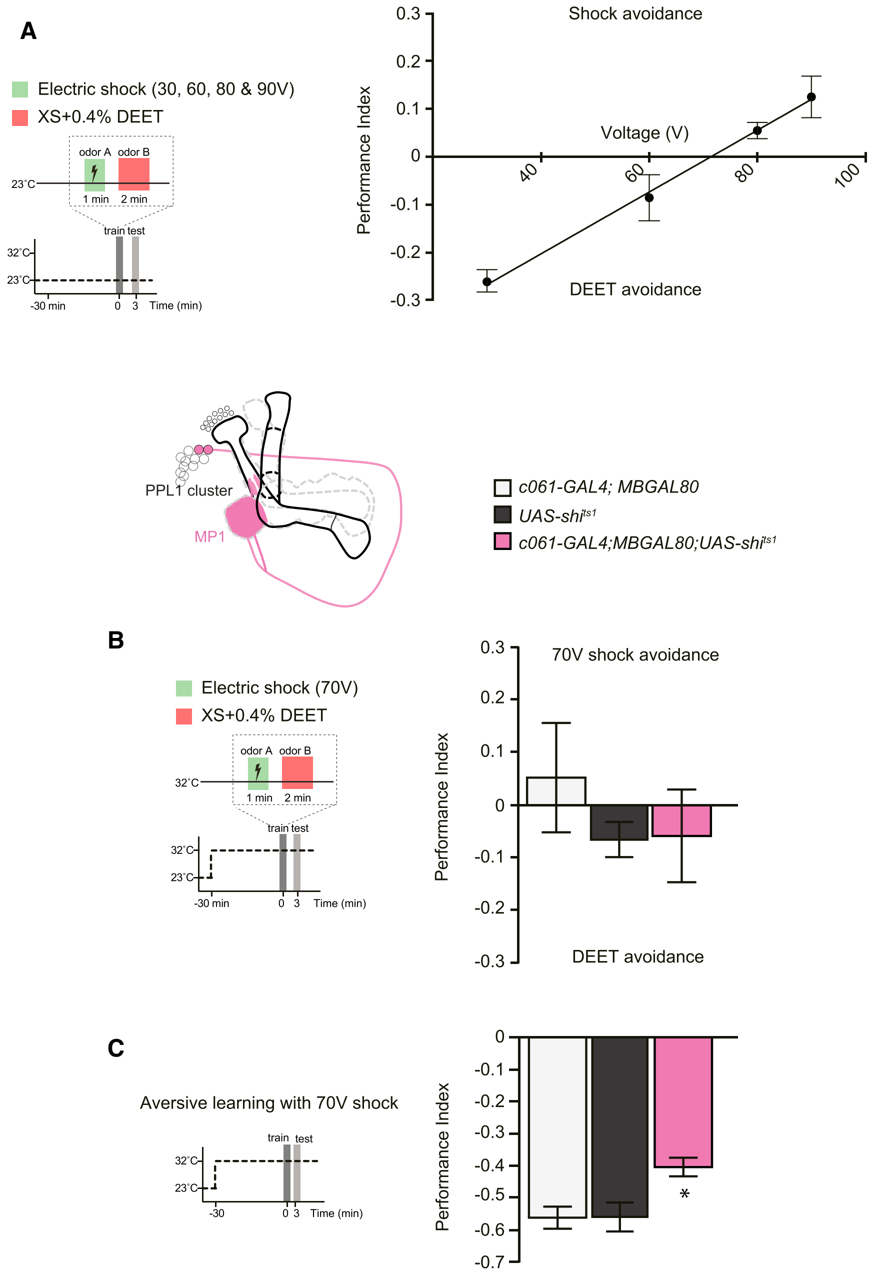

Figure 3. Discrete Aversive Signals Use Common Reinforcing Dopaminergic Neurons

(A) Flies were differentially conditioned by pairing of one odor with electric shock of varying magnitude and the other odor with 0.4% DEET in sugar carrier. They were then immediately tested for olfactory preference. Flies avoided the odor that had been previously paired with 80 or 90 V but preferred the odor if it was paired with 30 or 60 V. Linear regression suggested that the intersecting point of equivalence between DEET and shock reinforcement was ~70 V ($R^2 = 0.68$). $n \geq 8$.

(B) Blockade of MP1 neurons did not alter the equivalent value of 0.4% DEET and 70 V. Flies trained with 70 V versus 0.4% DEET showed no learned odor preference. Performance of c061; MBGAL80; UAS-*shi*^{ts1} flies with blocked MP1 neurons was indistinguishable from that of control groups ($p > 0.6$, ANOVA). None of the groups were statistically significant from zero ($p > 0.1$, one-sample t test, $n \geq 5$).

(C) Blockade of MP1 neurons partially impaired aversive learning with 70 V. Performance of c061; MBGAL80; UAS-*shi*^{ts1} flies was statistically different from that of control groups ($p < 0.05$, ANOVA, $n \geq 5$).

Data are shown as mean ± SEM.

DEET in 1 M sucrose showed a similar dose-dependent aversive learning to those trained in prior experiments with DEET in xylose and sucrose carrier, although the optimal DEET concentration for learning shifted from 0.4% to 0.6% (Figure 4A). We next tested the DEET memory performance of *Tbh*^{M18} mutant flies that are impaired in appetitive learning. Strikingly, whereas the behavior of wild-type flies became conditioned approach within 30 min, *Tbh*^{M18} flies showed a more persistent aversive memory performance, with scores remaining significantly negative 30 and 60 min after training (Figure 4B). However, the performance still converted from odor avoidance to approach by 24 hr. Since octopamine only provides short-term sweet-taste reinforcement [9], we hypothesized that persistent nutrient-dependent memory must be independently formed in *Tbh*^{M18} flies. Indeed, *Tbh*^{M18} flies trained with 1 M sucrose did not display immediate memory, but significant performance emerged 1 hr after training and remained for at least 24 hr (Figure 4C). These data support the prior model of octopamine specifically conveying short-term appetitive reinforcement and not the nutrient-dependent long-term signal [9]. In addition, they suggest that our DEET learning protocols form parallel aversive and appetitive memories. To further test a parallel memory trace model, we trained flies with 0.3% DEET and 1 M sucrose, a combination with which no immediate odor avoidance or approach performance is evident, and blocked either the rewarding or aversive dopaminergic neurons during training (Figures 4D–4F). Strikingly, blockade of the rewarding dopaminergic neurons with 0104; UAS-*shi*^{ts1} revealed significant conditioned avoidance (Figure 4E). In contrast, blockade of the negatively reinforcing MB-MP1 dopaminergic neurons with c061; MBGAL80; UAS-*shi*^{ts1} uncovered significant conditioned odor approach performance (Figure 4F). We therefore conclude that training with the compound DEET and sugar stimulus leads to the independent formation of aversive and appetitive memories. The differing stability of these competing memories subsequently determines which one of them guides learned behavior after training.

The extent to which rewarding and aversive stimuli are coded in mammalian dopaminergic neurons is hotly debated [2, 28, 29]. Recordings in the monkey have shown that some dopaminergic neurons respond to either bitter taste or an aversive air puff [30], suggesting that the quality of an aversive reinforcer may be represented. Work in flies has functionally split dopaminergic neurons into groups that are critical for reward

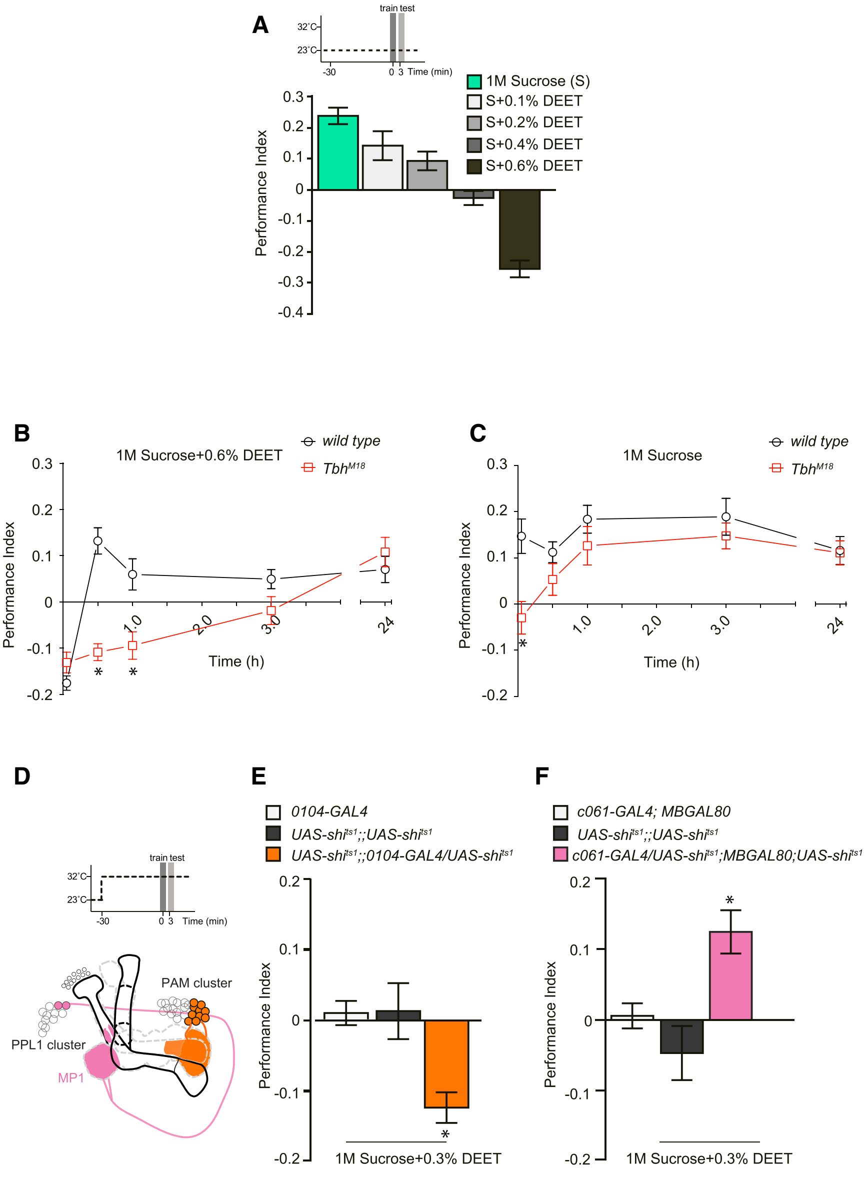

Figure 4. DEET:Sucrose Learning Forms Parallel Competing Appetitive and Aversive Memories

(A) Learning with DEET in 1 M sucrose depends on concentration. Wild-type flies starved overnight were trained with 0% to 0.6% DEET in 1 M sucrose. Increase of DEET concentration impaired appetitive memory performance and formed robust aversive performance when presented as 0.6%. Performance with 0.6% DEET is significantly different from that of all other groups (all $p < 0.0001$, ANOVA, $n \geq 10$).

(B) Aversive DEET memory is longer lasting in *Tbh*^{M18} mutant flies trained with 1 M sucrose plus 0.6% DEET. Wild-type flies exhibit robust aversive memory immediately after training, but the performance converts to stable and long-lasting conditioned approach 30 min later. In contrast, *Tbh*^{M18} mutant flies show significant aversive memory 30 and 60 min after training, compared to that of the wild-type flies (both $p < 0.005$, t test). Memory performance of the *Tbh*^{M18} mutant flies also converts to conditioned approach by 24 hr and was not significantly different from that of wild-type flies ($p > 0.1$, t test). All $n \geq 10$.

(C) *Tbh*^{M18} mutant flies lack short-term sweet-taste sucrose-reinforced memory but form long-term nutrition-dependent memory. Wild-type and *Tbh*^{M18} mutant flies were starved overnight and trained with 1 M sucrose in 1% agar. *Tbh*^{M18} flies showed significantly defective immediate memory performance ($p < 0.005$, t test) but were indistinguishable from wild-type flies 0.5, 1, 3, and 24 hr after training (all $p > 0.1$, t test). All $n \geq 8$.

(D) Training paradigm for testing the role of MP1 and PAM dopaminergic neurons in DEET learning. The mushroom body lobe innervation of each type of dopaminergic neuron is illustrated.

(E) Blockade of rewarding PAM dopaminergic neurons enhanced aversive memory performance after learning with 1 M sucrose plus 0.3% DEET. Performance of 0104-GAL4 UAS-*shi*^{ts1} flies was significantly different from that of both control groups ($p < 0.005$, ANOVA, $n \geq 15$).

(F) Blockade of the negatively reinforcing MP1 neurons enhanced appetitive memory performance after learning with 1 M sucrose plus 0.3% DEET. Performance of c061; MBGAL80; UAS-*shi*^{ts1} flies was significantly different from that of both control groups ($p < 0.005$, ANOVA, $n \geq 19$).

Data are shown as mean ± SEM.

learning and others for aversive learning [3, 5–9]. However, recent studies suggested a requirement for modulation of the aversive system in appetitive learning [9] and demonstrated a role for rewarding dopaminergic neurons in relative aversive learning [31]. In addition, imaging activity in negatively reinforcing MB-MP1 neurons revealed responses to both sweet sugar and bitter DEET. Nevertheless, the DEET reinforcement data presented here, when taken with published knowledge of shock reinforcement [7], imply that flies utilize the same, or at least an overlapping, evaluation system to convey the reinforcing effects of discrete aversive stimuli. It will be interesting to determine the respective input pathways to the negatively reinforcing dopaminergic neurons. These experiments also highlight the importance of being able to both record from and control recognizable subpopulations of dopaminergic neurons. Without intervention, it is difficult to understand whether a given dopaminergic neuron provides a reinforcement or motivational salience [2] signal.

Perhaps most surprisingly, our data demonstrate that during learning flies independently assign the value of individual components of a compound food stimulus to an odor. Rather than forming a single memory of the relative quality of the tainted sugar, they learn the bitter and sugar components in parallel. This multiplexing is further illustrated by sugars in which octopamine distinguishes between memories of sweet taste and nutrient components [9]. These results suggest that despite the integration of tastant information that occurs within the first layers of the gustatory system [15, 16, 32, 33] and provides control over food ingestion, each component also gains unprocessed access to the negative and positive arms of the reinforcement system. The fly therefore appears to retain as much information of foraging history as possible,

while allowing the relative persistence of the resultant constituent memories to inform later behavior. Such a mechanism might help the fly to direct short-term foraging away from food sources that happen to be unpalatable but remember that they are usually nutritious.

Experimental Procedures

Details of all experiments are provided in the Supplemental Experimental Procedures.

Fly Strains

Flies were raised on cornmeal food at 25°C and 40%–50% relative humidity. The wild-type Canton-S [23], c061; MBGAL80 [23], *Tbh*^{M18} [20], *dumb*^{1} [21], UAS-*shi*^{ts1} [22], R73F07-GAL4 [24], NP1528-GAL4 [6], NP5272-GAL4 [6], UAS-*dTrpA1* [19], *Gr66a*-GAL4 [34], UAS-*hid*:UAS-rpr [17], UAS-*GCaMP3* [26], *TH*-GAL4 [27], and UAS-*IR40a*^{RNAi} [12] flies have all been described.

Behavioral Analysis

Mixed-sex populations of 6- to 9-day-old flies were tested together in all experiments. For DEET training, groups of ~100 flies were food deprived for 22–28 hr in vials containing 2–3 ml 1% agar and filter paper. Liquid DEET was diluted to the appropriate final concentration with a given sugar solution in 1% molten agar (in water) and shaken to create a suspension and spread onto filter paper. Dried papers were rolled into training tubes. Training paradigms are indicated in the figure legends.

A performance index (PI) was calculated as the number of flies approaching (appetitive) or avoiding (aversive) the conditioned odor minus the number going the other direction, divided by the total number of flies in the experiment. A single PI is the average of two experiments in which identical genotype flies are trained with the reciprocal reinforced/non-reinforced odor combination. Odors were 3-octanol and 4-methylcyclohexanol.

Two-Photon In Vivo Calcium Imaging

Adult, food-deprived UAS-GCaMP3; THGAL4 flies were waxed to a custom-built imaging chamber, and the head capsule was removed under ice-cold sugar-free saline. Two-photon imaging was performed with a custom-made imaging setup as described [35].

Statistical Analysis

Statistical analyses were performed with PRISM (GraphPad Software) and are specifically referenced in the figure legends.

Supplemental Information

Supplemental Information includes Supplemental Experimental Procedures and three figures and can be found with this article online at http://dx.doi.org/10.1016/j.cub.2014.05.078.

Acknowledgments

We thank Kristin Scott, Hiromu Tanimoto, Gerry Rubin, and the Janelia Farm Project for flies and Wolf Huetteroth for the schematic artwork. We also thank members of the S.W. and Miesenböck labs for discussion. We especially thank Ruth Brain and Paola Cognigni for help with fly strains and other reagents. M.K. was funded by a Boehringer Ingelheim fellowship. E.P. thanks the Philippe and Bettencourt-Schueller Foundations. S.W. is supported by a Wellcome Trust Senior Research Fellowship in the Basic Biomedical Sciences and by funds from the Gatsby Charitable Foundation and Oxford Martin School.

Received: January 6, 2014
Revised: April 28, 2014
Accepted: May 21, 2014
Published: July 17, 2014

References

1. Schultz, W. (2010). Dopamine signals for reward value and risk: basic and recent data. Behav. Brain Funct. *6*, 24.
2. Bromberg-Martin, E.S., Matsumoto, M., and Hikosaka, O. (2010). Dopamine in motivational control: rewarding, aversive, and alerting. Neuron *68*, 815–834.
3. Waddell, S. (2013). Reinforcement signalling in Drosophila; dopamine does it all after all. Curr. Opin. Neurobiol. *23*, 324–329.
4. Schwaerzel, M., Monastirioti, M., Scholz, H., Friggi-Grelin, F., Birman, S., and Heisenberg, M. (2003). Dopamine and octopamine differentiate between aversive and appetitive olfactory memories in Drosophila. J. Neurosci. *23*, 10495–10502.
5. Claridge-Chang, A., Roorda, R.D., Vrontou, E., Sjulson, L., Li, H., Hirsh, J., and Miesenböck, G. (2009). Writing memories with light-addressable reinforcement circuitry. Cell *139*, 405–415.
6. Aso, Y., Siwanowicz, I., Bräcker, L., Ito, K., Kitamoto, T., and Tanimoto, H. (2010). Specific dopaminergic neurons for the formation of labile aversive memory. Curr. Biol. *20*, 1445–1451.
7. Aso, Y., Herb, A., Ogueta, M., Siwanowicz, I., Templier, T., Friedrich, A.B., Ito, K., Scholz, H., and Tanimoto, H. (2012). Three dopamine pathways induce aversive odor memories with different stability. PLoS Genet. *8*, e1002768.
8. Liu, C., Plaçais, P.Y., Yamagata, N., Pfeiffer, B.D., Aso, Y., Friedrich, A.B., Siwanowicz, I., Rubin, G.M., Preat, T., and Tanimoto, H. (2012). A subset of dopamine neurons signals reward for odour memory in Drosophila. Nature *488*, 512–516.
9. Burke, C.J., Huetteroth, W., Owald, D., Perisse, E., Krashes, M.J., Das, G., Gohl, D., Silies, M., Certel, S., and Waddell, S. (2012). Layered reward signalling through octopamine and dopamine in Drosophila. Nature *492*, 433–437.
10. Ditzen, M., Pellegrino, M., and Vosshall, L.B. (2008). Insect odorant receptors are molecular targets of the insect repellent DEET. Science *319*, 1838–1842.
11. Pellegrino, M., Steinbach, N., Stensmyr, M.C., Hansson, B.S., and Vosshall, L.B. (2011). A natural polymorphism alters odour and DEET sensitivity in an insect odorant receptor. Nature *478*, 511–514.
12. Kain, P., Boyle, S.M., Tharadra, S.K., Guda, T., Pham, C., Dahanukar, A., and Ray, A. (2013). Odour receptors and neurons for DEET and new insect repellents. Nature *502*, 507–512.
13. Lee, Y., Kim, S.H., and Montell, C. (2010). Avoiding DEET through insect gustatory receptors. Neuron *67*, 555–561.
14. Burke, C.J., and Waddell, S. (2011). Remembering nutrient quality of sugar in Drosophila. Curr. Biol. *21*, 746–750.
15. Wang, Z., Singhvi, A., Kong, P., and Scott, K. (2004). Taste representations in the Drosophila brain. Cell *117*, 981–991.
16. Thorne, N., Chromey, C., Bray, S., and Amrein, H. (2004). Taste perception and coding in Drosophila. Curr. Biol. *14*, 1065–1079.
17. Zhou, L., Schnitzler, A., Agapite, J., Schwartz, L.M., Steller, H., and Nambu, J.R. (1997). Cooperative functions of the reaper and head involution defective genes in the programmed cell death of Drosophila central nervous system midline cells. Proc. Natl. Acad. Sci. USA *94*, 5131–5136.
18. Marella, S., Fischler, W., Kong, P., Asgarian, S., Rueckert, E., and Scott, K. (2006). Imaging taste responses in the fly brain reveals a functional map of taste category and behavior. Neuron *49*, 285–295.
19. Hamada, F.N., Rosenzweig, M., Kang, K., Pulver, S.R., Ghezzi, A., Jegla, T.J., and Garrity, P.A. (2008). An internal thermal sensor controlling temperature preference in Drosophila. Nature *454*, 217–220.
20. Monastirioti, M., Linn, C.E.J., Jr., and White, K. (1996). Characterization of Drosophila tyramine beta-hydroxylase gene and isolation of mutant flies lacking octopamine. J. Neurosci. *16*, 3900–3911.
21. Kim, Y.C., Lee, H.G., and Han, K.A. (2007). D1 dopamine receptor dDA1 is required in the mushroom body neurons for aversive and appetitive learning in Drosophila. J. Neurosci. *27*, 7640–7647.
22. Kitamoto, T. (2001). Conditional modification of behavior in Drosophila by targeted expression of a temperature-sensitive shibire allele in defined neurons. J. Neurobiol. *47*, 81–92.
23. Krashes, M.J., DasGupta, S., Vreede, A., White, B., Armstrong, J.D., and Waddell, S. (2009). A neural circuit mechanism integrating motivational state with memory expression in Drosophila. Cell *139*, 416–427.
24. Jenett, A., Rubin, G.M., Ngo, T.T., Shepherd, D., Murphy, C., Dionne, H., Pfeiffer, B.D., Cavallaro, A., Hall, D., Jeter, J., et al. (2012). A GAL4-driver line resource for Drosophila neurobiology. Cell Rep. *2*, 991–1001.
25. Sitaraman, D., Zars, M., Laferriere, H., Chen, Y.C., Sable-Smith, A., Kitamoto, T., Rottinghaus, G.E., and Zars, T. (2008). Serotonin is necessary for place memory in Drosophila. Proc. Natl. Acad. Sci. USA *105*, 5579–5584.
26. Tian, L., Hires, S.A., Mao, T., Huber, D., Chiappe, M.E., Chalasani, S.H., Petreanu, L., Akerboom, J., McKinney, S.A., Schreiter, E.R., et al. (2009).

Imaging neural activity in worms, flies and mice with improved GCaMP calcium indicators. Nat. Methods *6*, 875–881.
27. Friggi-Grelin, F., Coulom, H., Meller, M., Gomez, D., Hirsh, J., and Birman, S. (2003). Targeted gene expression in Drosophila dopaminergic cells using regulatory sequences from tyrosine hydroxylase. J. Neurobiol. *54*, 618–627.
28. Schultz, W. (2010). Subjective neuronal coding of reward: temporal value discounting and risk. Eur. J. Neurosci. *31*, 2124–2135.
29. Fiorillo, C.D. (2013). Two dimensions of value: dopamine neurons represent reward but not aversiveness. Science *341*, 546–549.
30. Fiorillo, C.D., Yun, S.R., and Song, M.R. (2013). Diversity and homogeneity in responses of midbrain dopamine neurons. J. Neurosci. *33*, 4693–4709.
31. Perisse, E., Yin, Y., Lin, A.C., Lin, S., Huetteroth, W., and Waddell, S. (2013). Different kenyon cell populations drive learned approach and avoidance in Drosophila. Neuron *79*, 945–956.
32. Dethier, V.G. (1976). The Hungry Fly: A Physiological Study of the Behaviour (Cambridge: Harvard University Press).
33. Marella, S., Mann, K., and Scott, K. (2012). Dopaminergic modulation of sucrose acceptance behavior in Drosophila. Neuron *73*, 941–950.
34. Scott, K., Brady, R.J., Jr., Cravchik, A., Morozov, P., Rzhetsky, A., Zuker, C., and Axel, R. (2001). A chemosensory gene family encoding candidate gustatory and olfactory receptors in Drosophila. Cell *104*, 661–673.
35. Lin, A.C., Bygrave, A.M., de Calignon, A., Lee, T., and Miesenböck, G. (2014). Sparse, decorrelated odor coding in the mushroom body enhances learned odor discrimination. Nat. Neurosci. *17*, 559–568.

Current Biology, Volume *24*

Supplemental Information

Drosophila Learn Opposing Components of a Compound Food Stimulus

Gaurav Das, Martín Klappenbach, Eleftheria Vrontou, Emmanuel Perisse, Christopher M. Clark, Christopher J. Burke, and Scott Waddell

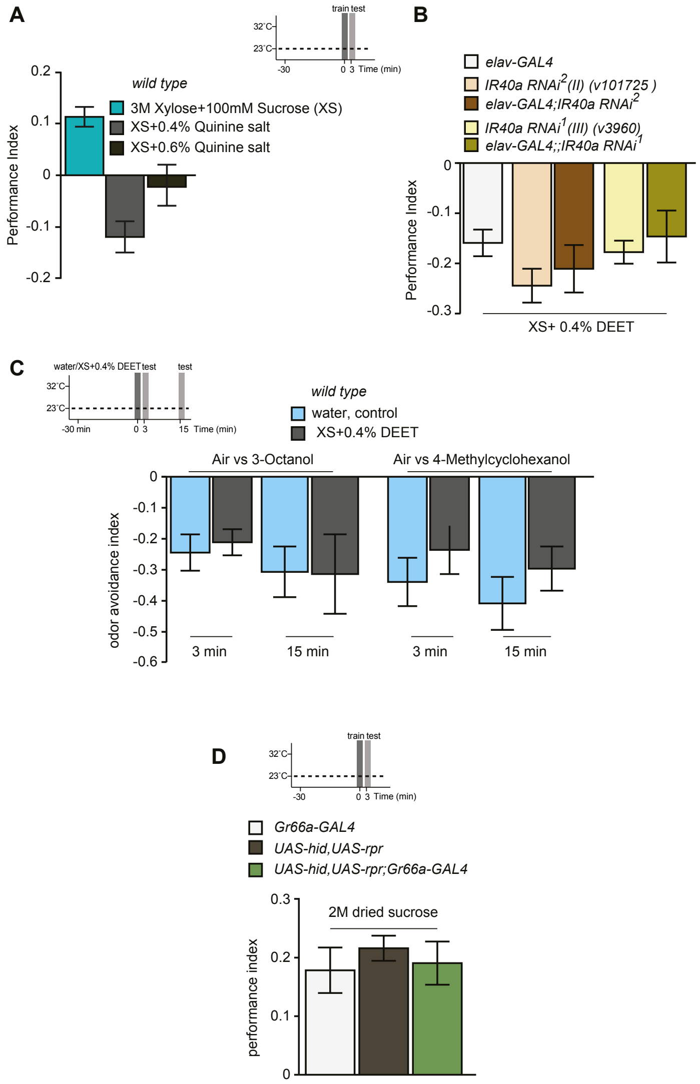

Figure S1. Aversive learning with bitter quinine and requirements for DEET learning. Related to Figure 1.

(A) Aversive learning with bitter quinine hydrochloride salt shows similar concentration dependence to learning with DEET. Wild type flies starved overnight were trained with 0.4 and 0.6% quinine salt in carrier sugars and were immediately tested for olfactory memory. Robust aversive memory was formed with 0.4% quinine but performance was negligible with 0.6%. n≥ 6.

(B) Learning with 0.4% DEET in sugar carrier is independent of the IR40a olfactory DEET receptor. Flies expressing either the v101725 or v3960 *IR40a* UAS-RNAi construct driven by *elav-GAL4* were trained with 0.4% DEET in sugar carrier. No significant difference in DEET learning was observed compared to control flies. (p>0.4, n≥ 3-6).

(C) DEET feeding does not alter olfactory acuity. Wild type flies fed 0.4% DEET in carrier or water for 2 min were tested immediately, or 15 min afterwards. No significant difference was observed between the relevant experimental and control groups, (all p>0.5, n≥ 4-6).

(D) Flies with ablated *Gr66a*-GAL4 bitter-sensing neurons are not impaired for sugar learning. No statistical differences were apparent between ablated flies and control groups (p>0.7, n≥ 4-6).

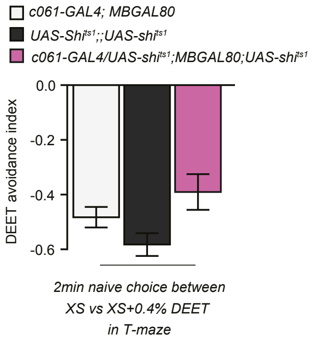

Figure S2. MP1 neurons are not required for DEET avoidance in naïve flies. Related to Figure 2.

Blocking the MP1 neurons with c061; MBGAL80; UAS-*shi*^{ts1} did not significantly impair naïve DEET avoidance. ($p>0.3$, except with UAS-*shi*^{ts1} $p=0.03$, ANOVA, $n= 8$).

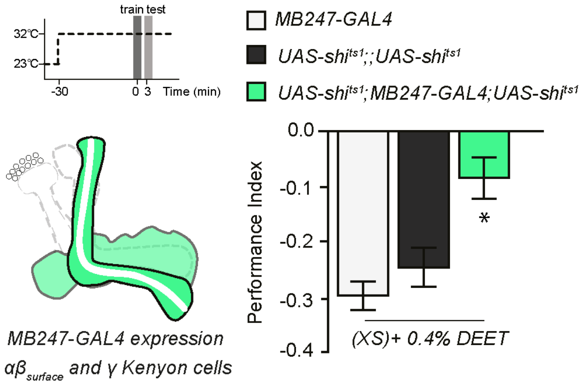

Figure S3. DEET memory performance requires mushroom body output. Related to Figure 2.

Blocking transmission from αβ surface and γ mushroom body neurons with MB247-GAL4 driven UAS-*shi*^{ts1} (X, III) significantly impaired DEET learning ($p < 0.005$, ANOVA, $n \geq 12$). Schematic depicts the experimental protocol and MB247-GAL4 expression pattern (green).

Supplemental Experimental Procedures

Fly strains

Fly stocks were raised on standard cornmeal food at 25°C and 40-50% relative humidity. The wild-type *Drosophila* strain used in this study is Canton-S. The MB-MP1 expressing c061;MBGAL80 flies are described. MBGAL80 suppresses GAL4 activity in mushroom body neurons in these flies [23]. Tbh^{M18} and $dumb^{1}$ mutant flies are described [20,21]. We used flies carrying UAS-shi^{ts1} on the third chromosome [UAS-shi^{ts1} (III)] or on the X and third chromosome [UAS-shi^{ts1} (X, III)] [22]. Flies expressing shi^{ts1} in MB-MP1 neurons were generated by crossing UAS-shi^{ts1} (X, III) (Fig. 2E) or UAS-shi^{ts1} (III) males (Fig. 2H, 3B and C) to homozygous c061;MBGAL80 females. Flies expressing shi^{ts1} in MB-MV1 or M3 neurons were generated by crossing UAS-shi^{ts1} (X and III) females to homozygous R73F07-GAL4 males [24] or NP1528-GAL4 and NP5272-GAL4 [6] males, respectively. MB247-GAL4 [S1] homozygous males were crossed to UAS-shi^{ts1} (X and III) females to drive shi^{ts1} expression in mushroom body neurons. Heterozygous control flies were generated by crossing UAS-shi^{ts1} (III or X and III) or GAL4 flies to wild-type flies. Flies expressing dTrpA1 in bitter taste neurons were established by crossing *UAS-dTrpA1* [19] females to homozygous *Gr66a*-GAL4 males [35]. To ablate Gr66a neurons, UAS-*hid*:UAS-rpr [17] flies were crossed to *Gr66a*-GAL4. To generate flies expressing *dTrpA1* in bitter taste neuron of $dumb^{1}$ flies, *UAS-dTrpA1;* $dumb^{1}$ females were crossed to *Gr66a*-GAL4; $dumb^{1}$ males. UAS-*GCaMP3* flies are described [26] and were crossed to *TH*-GAL4 flies [27] for *in vivo* Calcium imaging. We knocked down *IR40a* using UAS-RNAi lines [12] driven pan-neurally by *elav*-GAL4 (X).

Behavioral analysis

Mixed sex populations of 6-9 day old flies raised at 25°C were tested together in all behavior experiments. Prior to training flies with DEET, groups of ~100 flies were food-deprived for 22-28 h in vials containing 2-3 ml 1% agar and a strip of filter paper. For CS+ reinforcement, liquid DEET was diluted to a final concentration of 0.4% (or as otherwise stated) along with 3M Xylose and 100mM Sucrose, in 1% molten agar (in water), shaken well to create a suspension and ~3ml was rapidly spread in an even layer on a 50 X 70 mm piece of filter paper, backed with Parafilm. 1% agar in water was used for the CS-. After drying for 30-40 min, the papers were rolled into T-maze training tubes. For experiments at 32°C, the training papers were further dried for ~30 minutes at 32°C. For both 23°C and 32°C experiments, humidity was maintained below 55%.

Flies were trained by first exposing them to one odor with 1% agar for 2 min, then clear airflow for 30 s. They were then tapped into a training tube, lined with carrier sugar and DEET and exposed to a second odor for 2 min. To test 3 min memory, flies were immediately transferred to the T-maze and given 2 min to choose between the two odors (Figs 1A, C, D, 2A, E-H). For 15, 30 60 min, 3 h and 24 h memory testing, flies were transferred back to starvation tubes before being reloaded into the T-maze for testing (Fig 1D, 4B-C). For all conditioning experiments, Performance Index (PI) was calculated as the number of flies approaching (appetitive memory) or avoiding (aversive memory) the conditioned odor minus the number of flies going the other direction, divided by the total number of flies in the experiment. A single PI value is the average score from flies of the identical genotype tested with the reciprocal reinforced/non-reinforced odor combination. Odors used were 3-octanol (OCT, 7 µl in 8 ml mineral oil) and 4-methylcyclohexanol (MCH, 6-7 µl in 8 ml mineral oil).

To block specific neurons with UAS-shi^{ts1}, flies in vials were transferred to 32°C, according to the schematics accompanying each figure (Figs 2D 3B,C and 4D).

For memory implantation experiments using UAS-*dTrpA1* mediated neural

activation (Figs 1F and 2C) 8-11 day old flies raised at room temperature and starved overnight were presented with one odor at restrictive 32°C for 2 min. During the next 45 s, they were transferred into a training tube with the other odor at permissive 23°C and left for 2 min. Flies were tested for immediate memory, or returned to starvation vials for testing at 3 h.

Aversive shock memory (Fig 3C) was assayed as described [31,S2]. Before training groups of ~100 flies were housed for 18–20 h in a 25 ml vial containing standard cornmeal/agar food and a piece of filter paper. Reinforcement was 70V.

For differential aversive experiments (Fig 3A and B), flies were prepared as above for aversive memory and were conditioned as follows; 1 min one odor with twelve 30, 60, 70 or 90 V shocks at 5 s inter-stimulus interval (ISI), 45 s fresh air and followed by 2 min second odor with 0.4% DEET in carrier. They were immediately tested for memory performance.

For testing odor-avoidance post DEET feeding (Fig. S1C), flies were allowed 2 min to feed on 0.4% DEET in sugar carrier (3M xylose+100mM sucrose) and 1% agar, or just 1% agar inside T-maze training tubes. They were subsequently tested immediately or after 15 min for their preference between OCT/MCH and odor free airflow. An odor avoidance index was calculated, similar to PI value described above. Naïve DEET avoidance (Fig S2A) was assay in the T-maze by allowing flies 2 min to choose between a tube lined with sugar carrier or sugar carrier with DEET in 1% agar. A DEET avoidance index was calculated as for PI value.

Feeding assay

Feeding was measured as described [14,S3] with modifications. Sugar carrier (3M xylose +100mM sucrose) + varying concentration of DEET mixture was prepared in 1% agar with 0.4% FD&C Blue No. 1 dye. Flies were given 5 min to feed on the respective mixture accompanied by MCH/OCT odor flow and were then immediately frozen. 50 flies were then processed to release and isolate the consumed blue dye and absorbance was measured at 625 nm.

2-Photon *in vivo* calcium imaging

Adult, food deprived UAS-GCaMP3;THGAL4 flies were waxed to a custom built imaging chamber and the head capsule was removed under ice-cold sugar free saline (108 mM NaCl, 5 mM KCl, 8.2 mM $MgCl_2$, 4 mM $NaHCO_3$, 1 mM NaH_2PO_4, 15 mM Ribose, 5 mM HEPES, pH 7.5). Two-photon imaging was performed using a custom made imaging set-up as described [38]. In brief, fluorescence was excited using 140 fs pulses centered on 910 nm generated by a Ti-sapphire laser (Chameleon Ultra II, Coherent), attenuated by a Pockels cell (Conoptics 302RM). Brains were imaged using a Movable Objective Microscope (Sutter) with a Zeiss 203, 1.0 NA W-Plan-Apochromat objective. The microscope was controlled using MPScope 2.0 [S4] via a PCI-6110 DAQ board (National Instruments). Images were acquired at approximately 4 Hz. 0.4% DEET + 1M sucrose was delivered to the fly manually through a tubing while the fly was observed using a Stingray CCD camera (Allied Vision Technologies). Images were converted to Analyze format and motion corrected, when needed, by maximizing the pixel-by-pixel correlation between each frame and a reference frame. DF/F traces were calculated in MATLAB using manually drawn regions of interest (ROIs) for the background and brain structure of interest. Activity maps were generated from Gaussian-smoothed, background subtracted images. A baseline fluorescence image was calculated as the average over a 10 s prestimulus interval. Final traces were generated in Prism 6 (GraphPad Software).

Statistical Analysis

All statistical analyses were performed using PRISM (GraphPad Software) and tests are specifically referenced in the figure legends. For all T-maze behavioral experiments data is represented as mean ± standard error of the mean (SEM). Analysis of variance (ANOVA) was followed by planned pairwise comparisons between the relevant groups with a Tukey honestly significant difference HSD post hoc test. Listed p values are multiplicity adjusted. To determine whether values were significantly different form zero, one sample t-tests were performed. Linear regression was performed for Figure 3A (Y=0.006428*X-0.4592). In Figure 4B and C, multiple t-test was performed between the two groups at each time point, with Holm-Sidak correction for multiple comparisons.

Supplemental References

S1. Zars, T., Fischer, M., Schulz, R., and Heisenberg, M. (2000). Localization of a short-term memory in Drosophila. Science *288*, 672–675.

S2. Tully, T., and Quinn, W.G. (1985). Classical conditioning and retention in normal and mutant Drosophila melanogaster. J Comp Physiol [A]. *157*, 263-277.

S3. Tanimura, T., Isono, K., Takamura, T., and Shimada, I. (1982). Genetic dimorphism in the taste sensitivity to trehalose in Drosophila melanogaster. J. Comp. Physiol. A Neuroethol. Sens. Neural Behav. Physiol. *147*, 433-437.

S4. Nguyen, Q.T., Tsai, P.S., and Kleinfeld, D. (2006). MPScope: a versatile software suite for multiphoton microscopy. J. Neurosci. Methods. *156*, 351-359.
